# Risk factors for suicidal ideation and suicide attempt among medical students: A meta-analysis

**DOI:** 10.1371/journal.pone.0261785

**Published:** 2021-12-22

**Authors:** Chanhee Seo, Christina Di Carlo, Selina Xiangxu Dong, Karine Fournier, Kay-Anne Haykal

**Affiliations:** 1 Faculty of Medicine, University of Ottawa, Ottawa, Ontario, Canada; 2 Health Sciences Library, University of Ottawa, Ottawa, Ontario, Canada; Faculty of Medicine, SERBIA

## Abstract

**Background:**

Medical training poses significant challenge to medical student wellbeing. With the alarming trend of trainee burnout, mental illness, and suicide, previous studies have reported potential risk factors associated with suicidal behaviours among medical students. The objective of this study is to provide a systematic overview of risk factors for suicidal ideation (SI) and suicide attempt (SA) among medical students and summarize the overall risk associated with each risk factor using a meta-analytic approach.

**Methods:**

Systemic search of six electronic databases including MEDLINE, Embase, Education Source, Scopus, PsycInfo, and CINAHL was performed from database inception to March 19, 2021. Studies reporting original quantitative or epidemiological data on risk factors associated with SI and SA among undergraduate medical students were included. When two or more studies reported outcome on the same risk factor, a random-effects inverse variance meta-analysis was performed to estimate the overall effect size.

**Results:**

Of 4,053 articles identified, 25 studies were included. Twenty-two studies reported outcomes on SI risk factors only, and three studies on both SI and SA risk factors. Meta-analysis was performed on 25 SI risk factors and 4 SA risk factors. Poor mental health outcomes including depression (OR 6.87; 95% CI [4.80–9.82] for SI; OR 9.34 [4.18–20.90] for SA), burnout (OR 6.29 [2.05–19.30] for SI), comorbid mental illness (OR 5.08 [2.81–9.18] for SI), and stress (OR 3.72 [1.39–9.94] for SI) presented the strongest risk for SI and SA among medical students. Conversely, smoking cigarette (OR 1.92 [0.94–3.92]), family history of mental illness (OR 1.79 [0.86–3.74]) and suicidal behaviour (OR 1.38 [0.80–2.39]) were not significant risk factors for SI, while stress (OR 3.25 [0.59–17.90]), female (OR 3.20 [0.95–10.81]), and alcohol use (OR 1.41 [0.64–3.09]) were not significant risk factors for SA among medical students.

**Conclusions:**

Medical students face a number of personal, environmental, and academic challenges that may put them at risk for SI and SA. Additional research on individual risk factors is needed to construct effective suicide prevention programs in medical school.

## Introduction

“Put on your own oxygen mask before assisting others.”

- Randy Pausch, *The Last Lecture*

The physician burnout and suicide epidemic represents a major public health crisis [1]. The reported prevalence of burnout exceeds 50% of practicing physicians in the United States, a rate almost double that of the general population [2]. A meta-analysis of 25 studies estimated the suicide rate among male physicians to be 40% higher than the general population, and for female physicians, 130% higher [3]. Compared to individuals with a professional or doctoral degree in other disciplines, physicians work significantly longer hours, experience higher rates of emotional exhaustion and burnout, and report greater dissatisfaction with work-life balance [4]. These challenges have taken a toll on physicians, and continue to do so.

With the greater awareness of the ominous state of physician burnout and suicide, the spotlight has turned to the high levels of psychological distress, burnout, depression, and suicidality among medical students. In the United States, 56% of medical students report experiencing burnout while in medical school [5]. A recent meta-analysis reported the global prevalence of depression among medical students to be 27.2%, a disquieting figure that hovers over threefold in comparison to their peers of similar age [6]. Importantly, recent estimates of the global prevalence of suicidal ideation (SI) and suicide attempt (SA) among medical students suggested that 11.1% had thoughts of suicide, and 1.6% had attempted suicide in the preceding year [6,7].

Confronted with the alarming state of medical student well-being, several institutional bodies including medical schools have proposed and implemented various suicide prevention and wellness programs (e.g., resilience, mindfulness, stress management) [8–11]. However, the targeted risk factors and overall quality of these curricula are divergent, and little is known about their efficacy.

To begin understanding the factors contributing to medical student distress and how we can address them, it is important to first identify the risk factors for SI and SA among medical students. Despite a large body of literature demonstrating the alarming state of medical student well-being, there remains a paucity in a systematic overview and meta-analysis of the reported risk factors to date. The purpose of this paper is to provide a comprehensive overview of the risk factors for SI and SA among medical students and summarize the overall risk associated with each risk factor using a meta-analytic approach. From this analysis, we explore potential suicide prevention strategies and identify areas of focus for future research, suicide risk screening, prevention, and intervention programs in undergraduate medical education.

## Methods

### Protocol registration

A prospectively developed systematic review protocol was registered in the International Prospective Register of Systematic Reviews (PROSPERO, CRD42021242386). This study was carried out in accordance with the PRISMA reporting guidelines (S1 Table) [12].

### Changes from review protocol

In the original review protocol published in PROSPERO, we planned to use the Newcastle-Ottawa Scale (NOS) to assess the risk of bias of included studies. After initial screening of the literature, a change was made to the risk assessment tool as outlined below, as most studies included in this review were cross-sectional studies. As the NOS tool most effectively assesses the risk of bias in non-randomized cohort or case-control studies [13], the authors in consensus have decided to implement the Joanna Briggs Institute (JBI) Critical Appraisal Checklist for Cross-Sectional Studies for the purpose of this review [14].

### Search method

A comprehensive systematic search of published journal articles was performed by an information specialist (K.F.) using MEDLINE, Embase, CINAHL, APA PsycInfo, Education Source, and Scopus on October 18, 2020 and updated on March 19, 2021. The search strategy consisted of a combination of subject headings and keywords including “medical student”, “suicide”, “suicide ideation”, and “suicide attempt” and adapted for use in each database (S2 Table). We also performed a manual search of the bibliographies of eligible articles for potential inclusion.

### Eligibility criteria

We included peer-reviewed primary research articles based on original quantitative or epidemiological data on undergraduate medical students with SI or SA. Studies with cross-sectional, observational, longitudinal, cohort, and case-control designs were included in the study. Only studies that examined risk factors associated with medical students experiencing SI or SA during undergraduate medical training were included. Lastly, only studies reporting appropriate control or comparison group of medical students (i.e., not students of other discipline, or the general public) were considered.

We omitted studies that (1) failed to report appropriate control or comparison groups composed of medical students; (2) used qualitative or ecological design; (3) reported risk factors associated with lifetime SI or SA; (4) only reported prevalence of SI or SA; or (5) reported risk factors for SI or SA in other populations (e.g., college students in other disciplines). We excluded commentaries, case studies, reviews, editorials, letters to the editor, or opinion articles. There were no restrictions imposed on the language, location, or year of publication.

### Study selection and data extraction

We used Covidence (Veritas Health Information, Melbourne, Australia) as the primary screening tool for this review. Using this tool, we split the extracted articles among three reviewers (C.S., C.D.C., and S.D.) such that each article was screened independently by two of the three reviewers in duplicate. Studies were selected for inclusion using a two-step screening process performed by two independent reviewers. First, all studies retrieved from the database search underwent title and abstract screening to determine suitability based on the aforementioned eligibility criteria. Studies that were deemed potentially eligible were subsequently scrutinized for inclusion in full-text screening. In both steps, inter-rater calibration with 20 randomly selected references was performed to ensure screening accuracy.

Using a standardized data extraction table, 3 of the authors (C.S., C.D.C., and S.D.) extracted data on study characteristics, sample demographics, and risk factor outcomes (i.e., number of samples in exposure vs. non-exposure groups) or effect estimates (i.e., odds ratios). Any discrepancies in study selection or data extraction were resolved by consensus.

### Risk of bias assessment

Overall methodological quality of the included studies was evaluated using the Joanna Briggs Institute (JBI) Critical Appraisal Checklist [14]. Studies were rigorously appraised by two independent reviewers (C.S., C.D.C. or S.D.) and any discrepancies were resolved by consensus.

### Analysis of studies

When at least two studies reported data on the same risk factor, a random effects inverse variance meta-analysis was performed to estimate the overall effect of the identified risk factor using odds ratio (OR). When studies only reported overall or adjusted effect estimates for variables of interest, or in case summary data (e.g., numbers of events and participants) could not be extracted, data were pooled as generic inverse variance outcomes and analyzed using random effects meta-analysis. We also assessed for potential publication bias in relation to funnel plots [15]. The plots were first examined visually to assess for bias or systematic heterogeneity. When more than 10 studies reported data on the same risk factor, Egger’s test of regression and Begg & Mazumdar’s rank correlation test were performed to determine the significance of funnel plot asymmetry [15,16]. Considering the power to detect bias is low with smaller number of studies (i.e., <10 studies) [17], gender was the only SI risk factor with sufficient data that was suitable for assessment. The degree of heterogeneity was analyzed using the Cochrane *I*^*2*^ statistics with the following thresholds: low (0–40%), moderate (30–60%), substantial (50–90%), and considerable (75–100%) [18]. When there was considerable heterogeneity (i.e., *I*^*2*^ >75%), a leave-1-out meta-analysis was performed to elucidate the effect of each individual study on the overall estimate. Statistical significance was set *a priori* to *p* <0.05. Egger’s test of regression and Begg & Mazumdar’s rank correlation test were performed using the metafor package (version 2.1–0) in R software. Meta-analysis was performed using the Cochrane Collaboration’s Review Manager software (version 5.4).

## Results

### Search results

**Fig 1:**
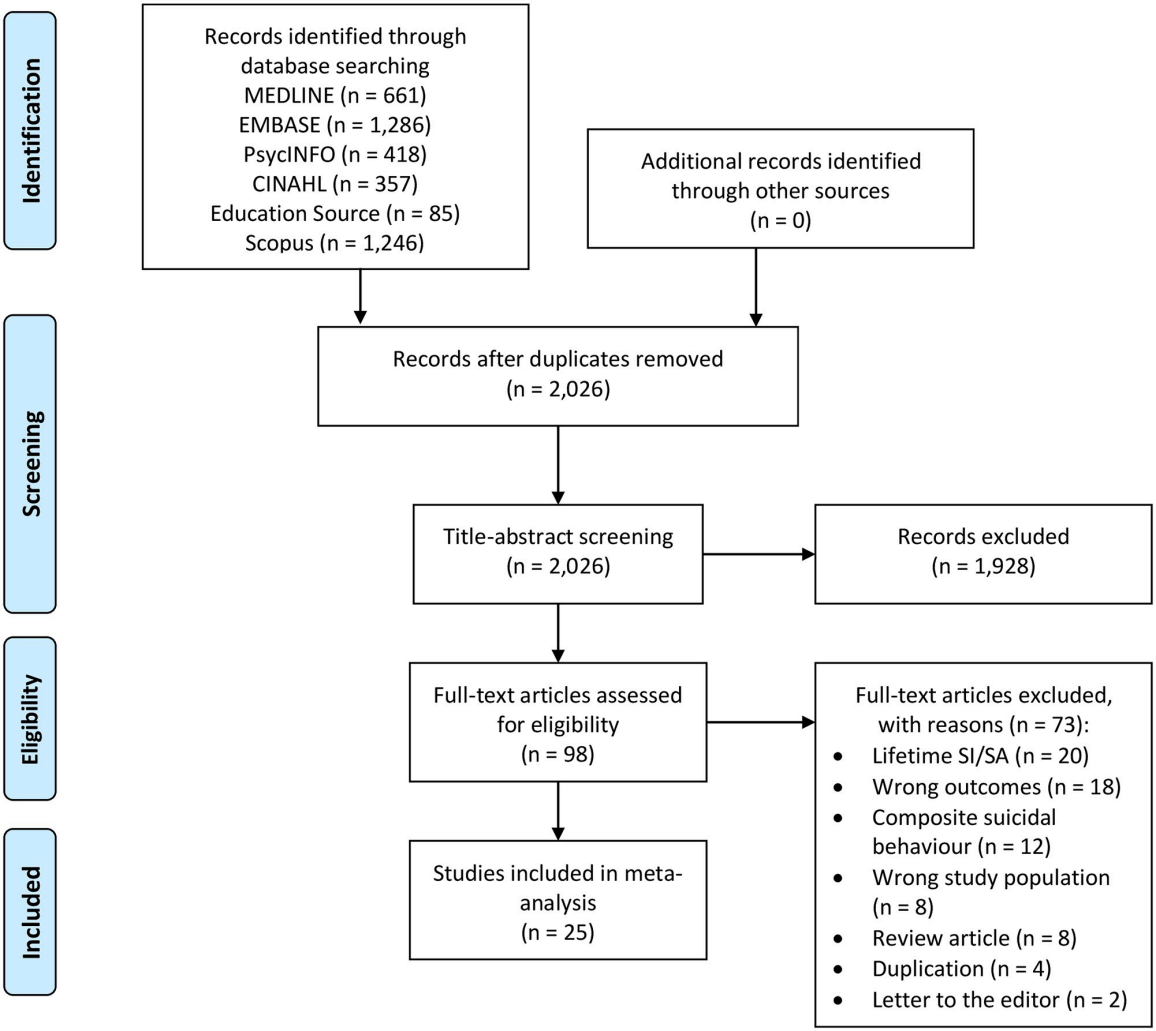
PRISMA flow diagram of the study screening process.

The electronic search of the databases retrieved a total of 4,053 references (Fig 1). After removing 2,027 duplicates, 2,026 references were identified for title-abstract screening. Of 98 full-text articles assessed for eligibility, 73 were excluded. No additional studies were identified in the manual bibliography search. In total, 31 studies met the inclusion criteria for this review, of which 25 studies provided sufficient data for meta-analysis.

### Study and participant characteristics

The characteristics of the included studies are summarized in Table 1. Twenty-four studies used cross-sectional methodology and one study used longitudinal survey methodology. In total, 26,111 participants were included. Twenty-two studies reported data on SI only, and three studies on both SI and SA. Studies examining the risk factors for SI and SA among medical students were published predominantly in the past decade (20/25 studies), of which the vast majority were in the last 5 years (16 studies). Studies were mainly conducted in South/Southeast Asia (8 studies) and North America (7 studies), followed by Middle East (5 studies), East Asia (2 studies), South Africa (2 studies), and South America (1 studies).

**Table 1:** Study characteristics.

Reference	Country	Study period	Study design	Level of training	Sample size	SI/SA		RoB score
						SI	SA	
Ahmed et al., 2016 [19]	Egypt	2016	CS	1^st^– 5^th^ year	612	√		7
Asfaw et al., 2020 [20]	Ethiopia	2019	CS	1^st^– 6^th^ year	710	√	√	8
Compton et al., 2008 [21]	US	1999–2003	LS	1^st^– 4^th^ year	2316	√		8
Desai et al., 2021 [22]	India	2017	CS	1^st^– 4^th^ year	506	√		8
Desalegn et al., 2020 [23]	Ethiopia	2019	CS	1^st^– 5^th^ year	393	√	√	8
Dyrbye et al., 2008 [5]	US	2006–2007	CS	1^st^– 4^th^ year	2248	√		7
Dyrbye et al., 2012 [24]	US	2009	CS	1^st^– 4^th^ year	2682	√		6
Ghazanfar et al., 2015 [25]	Pakistan	2012–2014	CS	1^st^– 5^th^ year	1132	√		5
Goebert et al., 2009 [26]	US	2003–2004	CS	1^st^– 4^th^ year	1215	√		7
Jackson et al., 2016 [27]	US	2012	CS	1^st^– 4^th^ year	4402	√		8
Khosravi et al., 2020 [28]	Iran	2018	CS	2^nd^– 7^th^ year	376	√		8
Madadin et al., 2020 [29]	Saudi Arabia	NR	CS	3^rd^– 6^th^ year	265	√		7
Maser et al., 2019 [30]	Canada	2015–2016	CS	1^st^– 4^th^ year	4613	√		8
Menezes et al., 2012 [31]	Nepal	2010	CS	1^st^– 4^th^ year	206	√		7
Okasha et al., 1981 [32]	Egypt	1978–1979	CS	5^th^ year	516	√		4
Osama et al., 2014 [33]	Pakistan	2013	CS	1^st^– 5^th^ year	331	√		6
Pham et al., 2019 [34]	Vietnam	2015–2016	CS	4^th^– 6^th^ year	494	√		7
Schwenk et al., 2010 [35]	US	2009	CS	1^st^– 4^th^ year	505	√		8
Nesan et al., 2020 [36]	India	2017	CS	NR	415	√		5
Sobowale et al., 2014 [37]	China	2012	CS	2^nd^– 3^rd^ year	348	√		4
Talih et al., 2018 [38]	Lebanon	2016	CS	1^st^– 4^th^ year	176	√		8
Tang et al., 2020 [39]	China	2015	CS	1^st^– 4^th^ year	662	√	√	6
Tareq et al., 2020 [40]	Bangladesh	2017	CS	3^rd^ year	399	√		4
Torres et al., 2018 [41]	Brazil	2011	CS	1^st^– 6^th^ year	475	√		8
Yousaf et al., 2016 [42]	Pakistan	2016	CS	2^nd^ year	114	√		4

Abbreviation: CS = cross-sectional survey; LS = longitudinal study; NR = not reported; RoB = risk of bias; SI = suicidal ideation; SA = suicide attempt; US = United States.

### Suicidal ideation risk factors

**Fig 2:**
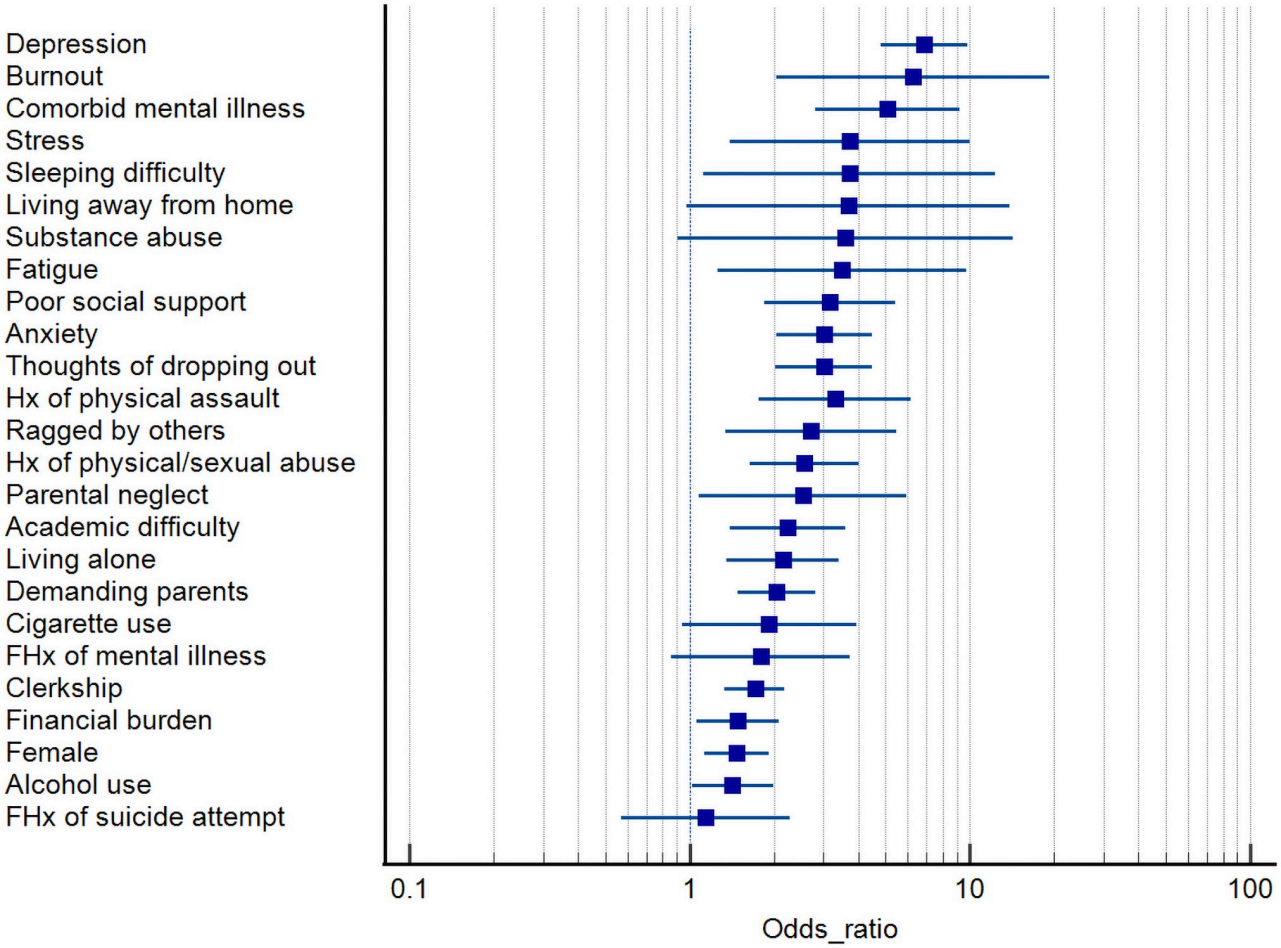
Summary forest plot of pooled effect estimates of risk factors for SI among medical students.

In total, 25 potential risk factors for suicidal ideation were identified by two or more studies and pooled by meta-analysis. The overall effect estimates are collated and summarized in Table 2 and visually represented in Fig 2. Detailed forest plots for each risk factor can be found in S1 Fig.

**Table 2:** Pooled effect estimates for risk factors associated with SI and SA among medical students.

**SI Risk factor**	**# of studies reporting outcome**	**Sample size**	**Odds ratio [95% CI]**	**P value**	***I* ^2^**
Academic difficulty	5 studies [22,29,31,34,41]	1946	2.23 [1.39, 3.58]	0.0009	30%
Alcohol use	7 studies [20,22,23,27,31,34,42]	7233	1.42 [1.02, 1.98]	0.04	54%
Anxiety	2 studies [20,41]	1232	3.02 [2.04, 4.47]	<0.00001	0%
Burnout	2 studies [5,38]	2424	6.29 [2.05, 19.30]	0.001	69%
Cigarette use	5 studies [22,23,31–33]	1952	1.92 [0.94, 3.92]	0.07	44%
Clerkship	8 studies [5,19,22,26,30,31,35,39]	10567	1.71 [1.33, 2.18]	<0.0001	50%
Comorbid mental illness	6 studies [22–24,33,36,41]	4802	5.08 [2.81, 9.18]	<0.00001	61%
Demanding parents	3 studies [25,33,36]	1878	2.04 [1.48, 2.81]	<0.0001	0%
Depression	8 studies [5,20,23,25,34,35,37,42]	6352	6.87 [4.80, 9.82]	<0.00001	69%
Fatigue	2 studies [25,39]	1794	3.49 [1.26, 9.71]	0.02	83%
Female	17 studies [5,19–23,28–31,33–35,39–42]	15172	1.47 [1.13, 1.92]	0.004	78%
FHx of mental illness	3 studies [20,23,25]	2282	1.79 [0.86, 3.74]	0.12	71%
FHx of suicide attempt	2 studies [20,22]	1263	1.14 [0.57, 2.27]	0.70	0%
Financial burden	4 studies [5,25,34,39]	4536	1.48 [1.06, 2.07]	0.02	55%
Hx physical/sexual abuse	3 studies [22,29,31]	977	2.57 [1.64, 4.02]	<0.0001	0%
Hx physical assault	2 studies [33,36]	746	3.30 [1.77, 6.14]	0.0002	0%
Living alone	3 studies [34,36,41]	1384	2.15 [1.36, 3.39]	0.0010	0%
Living away from home	5 studies [19,22,34,36,41]	2502	3.68 [0.98, 13.84]	0.05	94%
Parental neglect	4 studies [29,31,33,36]	1217	2.53 [1.08, 5.92]	0.03	71%
Poor social support	4 studies [20,23,25,41]	2757	3.15 [1.84, 5.41]	<0.0001	47%
Ragged by others	2 studies [31,33]	536	2.71 [1.34, 5.47]	0.006	0%
Sleeping difficulty	2 studies [25,32]	1648	3.72 [1.12, 12.35]	0.03	75%
Stress	4 studies [20,21,31,39]	3941	3.72 [1.39, 9.94]	0.009	92%
Substance abuse	4 studies [22,27,31,33]	5445	3.60 [0.91, 14.25]	0.07	77%
Thoughts of dropping out	3 studies [25,35,41]	2112	3.01 [2.03, 4.47]	<0.00001	0%
**SA Risk factor**	**Studies reporting outcome**	**Sample size**	**Odds ratio [95% CI]**	**P value**	***I*** ^**2**^
Alcohol use	2 studies [20,23]	1150	1.41 [0.64, 3.09]	0.39	0%
Depression	2 studies [20,23]	1150	10.34 [7.82, 13.67]	<0.00001	0%
Female	3 studies [20,23,39]	1812	3.20 [0.95, 10.81]	0.06	87%
Stress	2 studies [20,39]	1419	3.25 [0.59, 17.90]	0.18	80%

Abbreviation: CI = confidence interval; FHx = family history; Hx = history; SA = suicide attempt; SI = suicidal ideation.

#### Gender

Female gender was significantly associated with increased risk of SI among medical students compared with male counterparts (17 studies; OR 1.47; 95% CI [1.13–1.92]; *P* = 0.004; *I*^*2*^ = 78%). Even when heterogeneity was controlled by excluding one outlier study [23] in a leave-1-out meta-analysis, female gender remained as a significant risk factor for SI (OR 1.31 [1.08–1.59]; *P* = 0.006; *I*^*2*^ = 53%).

#### Relationship with parents

Previous experience of being neglected by parents was significantly associated with higher risk of SI among medical students (4 studies; OR 2.53 [1.08–5.92]; *P* = 0.03; *I*^*2*^ = 71%). The same was true for medical students who had demanding parents (3 studies; OR 2.04 [1.48–2.81]; *P*<0.0001; *I*^*2*^ = 0%).

#### Living situation

Medical students who lived alone were more likely to have SI than medical students who lived at home or with roommates (3 studies; OR 2.15 [1.36–3.39]; *P* = 0.0010; *I*^*2*^ = 0%). Conversely, while living away from home only trended towards higher SI risk (5 studies; OR 3.68 [0.98–13.84]; *P* = 0.05; *I*^*2*^ = 94%), when heterogeneity was controlled by excluding one outlier study [19], the risk of SI became statistically significant (OR 2.04 [1.14–3.64]; *P* = 0.02; *I*^*2*^ = 53%).

#### Substance use and abuse

Alcohol consumption was associated with increased risk of SI among medical students (7 studies; OR 1.42 [1.02–1.98]; *P* = 0.04; *I*^*2*^ = 54%). Conversely, smoking cigarette was not a significant risk factor for SI (5 studies; OR 1.92 [0.94–3.92]; *P* = 0.07; *I*^*2*^ = 44%). Although the association between substance abuse and SI only trended towards significance (4 studies; OR 3.60 [0.91–14.25]; *P* = 0.07; *I*^*2*^ = 77%), when heterogeneity was corrected by excluding one outlier study, [27] the new effect estimate reached statistical significance (OR 6.59 [1.15–37.85]; *P* = 0.03, *I*^*2*^ = 64%).

#### Past personal or family experience

Personal history of physical or sexual abuse (3 studies; OR 2.57 [1.64–4.02]; *P*<0.0001; *I*^*2*^ = 0%) and physical assault (2 studies; OR 3.30 [1.77–6.14]; *P* = 0.0002; *I*^*2*^ = 0%) were significantly associated with SI among medical students. Medical students who were ragged or humiliated by others also had greater risk of SI (2 studies; OR 2.71 [1.34–5.47]; *P* = 0.006; *I*^*2*^ = 0%).

Conversely, family history of mental illness (3 studies; OR 1.79 [0.86–3.74]; *P* = 0.12; *I*^*2*^ = 71%) and suicide attempt (2 studies; OR 1.14 [0.57–2.27]; *P* = 0.70; *I*^*2*^ = 0%) were not associated with SI risk.

#### Medical school environment

Compared with pre-clerkship students, clinical or clerkship students were more likely to engage in SI (8 studies; OR 1.71 [1.33–2.18]; *P*<0.0001; *I*^*2*^ = 50%). The same was true for medical students experiencing academic difficulty (5 studies; OR 2.23 [1.39–3.58]; *P* = 0.0009; *I*^*2*^ = 30%). Medical students with poor social support (4 studies; OR 3.15 [1.84–5.41]; *P*<0.0001; *I*^*2*^ = 47%) or those who were thinking of dropping out of medical school (3 studies; OR 3.01 [2.03–4.47]; *P*<0.00001; *I*^*2*^ = 0%) were more likely to have SI. The same was true for medical students from low-income family or in substantial debt (4 studies; OR 1.48 [1.06–2.07]; *P* = 0.02; *I*^*2*^ = 55%).

#### Mental health

Medical students who had comorbid mental illness were more likely to engage in SI (6 studies; OR 5.08 [2.81–9.18]; *P*<0.00001; *I*^*2*^ = 61%). The same was true for depression (8 studies; OR 6.87 [4.80–9.82]; *P*<0.00001; *I*^*2*^ = 61%), anxiety (2 studies; OR 3.02 [2.04–4.47]; *P*<0.00001; *I*^*2*^ = 0%), burnout (2 studies; OR 6.29 [2.05–19.30]; *P* = 0.001; *I*^*2*^ = 69%), and sleep difficulty (2 studies; OR 3.72 [1.12–12.35]; *P* = 0.03; *I*^*2*^ = 75%). Two studies identified fatigue as a risk factor for SI although there was considerable heterogeneity (OR 3.49 [1.26–9.71]; *P* = 0.02; *I*^*2*^ = 83%). Stress was also significantly associated with SI (4 studies; OR 3.72 [1.39–9.94]; *P* = 0.009; *I*^*2*^ = 92%), however no outlier study could be identified in a leave-1-out meta-analysis.

### Suicide attempt

**Fig 3:**
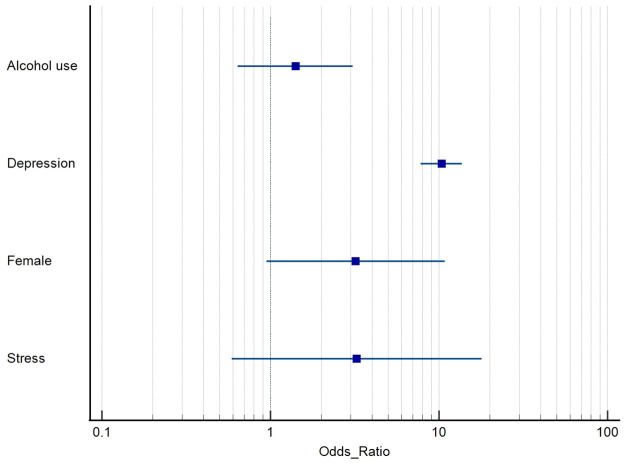
Summary forest plot of pooled effect estimates of risk factors for SA among medical students.

In total, 4 risk factors for suicide attempt were identified by two or more studies and pooled by meta-analysis. The overall effect estimates are collated and summarized in Table 2 and visually represented in Fig 3. Detailed forest plots for each risk factor can be found in S2 Fig.

#### Gender

Female gender as a risk factor for SA among medical students only trended towards significance (3 studies, OR 3.20 [0.95–10.71]; *P* = 0.06; *I*^*2*^ = 87%), and remained statistically non-significant even when one outlier study [23] was excluded from the analysis (OR 1.81 [0.90–3.61]; *P* = 0.09; *I*^*2*^ = 0%).

#### Substance use

Alcohol consumption (2 studies; OR 1.41 [0.64–3.09]; *P* = 0.39; *I*^*2*^ = 0%) was not associated with SA among medical students.

#### Mental health

Depression (2 studies; OR 10.34 [7.82–13.67]; *P*<0.00001; *I*^*2*^ = 0%) was significantly associated with increased risk of SA among medical students. Conversely, stress (2 studies; OR 3.25 [0.59–17.90]; *P* = 0.18; *I*^*2*^ = 80%) was not significantly associated with SA among medical students.

### Assessment of risk of bias and publication bias

Individual quality appraisal and risk of bias assessment scores are reported in Table 1. The maximum attainable score was 8. Overall, the mean score across the 25 studies was 6.65 ± 1.45. Furthermore, as illustrated by the funnel plot (S3 Fig), and statistically assessed by Egger’s test of regression (*P* = 0.410) and Begg & Mazumdar’s rank correlation test (*P* = 0.564), there was no evidence of significant publication bias.

## Discussion

This study aimed to provide a systematic overview of all risk factors pertaining to medical student SI and SA in the literature. To our knowledge, this is the largest and most comprehensive meta-analysis on risk factors for SI and SA among medical students performed to date. The meta-analysis of the various risk factors included results from 25 studies and identified that medical students with poor mental health outcomes, in the order of depression, burnout, comorbid mental illness, and stress, were at the highest risk for SI. Similar trends emerged with risk factors for SA among medical students, with depression representing the greatest risk.

When interpreting these findings, it is important to contextualize the results against the general population as a number of the reported risk factors identified in this review did not pertain only to medical education. For instance, comparing the results from our study to a meta-analysis on the association of depressive symptoms and SI among Chinese university students, medical students with depression were more likely to report SI [43]. Similarly, medical students endorsing psychological distress, when compared to the general population, were more likely to experience SI [44]. Conversely, female gender [45], anxiety [46], alcohol use [47], and substance abuse [48] as risk factors for SI among medical students were comparable to the reported findings from the general population. Although firm conclusions cannot be drawn from such comparisons, together, findings of our study suggest that medical students share some common risk factors for SI and SA as the general population, some of which may be more strongly associated in comparison to the public. Future studies would benefit from conducting direct statistical comparisons using validated scales to establish unique risk factors pertinent to medical students versus the general population.

Our study also highlights the importance of certain living arrangements as a predictor of SI among medical students. For example, medical students who live alone or live away from home were at increased risk for SI compared to those who live with roommates, classmates, or parents. While the present study cannot provide a conclusive explanation of the association between such living arrangements and SI, these findings offer some support to previous research that highlighted deficiencies in one’s social network as a significant risk for SI, potentially mediated by depression and anxiety [49,50]. Similarly, our findings corroborate that medical students with poor social support were more likely to have SI. Taken together, these findings propound that provision of strong social support should be the cornerstone for suicide prevention and student wellness programs in medical school.

Medical students also face a unique set of stressors and strains that extend beyond the academic curricula in medical school. As students adjust to the medical school environment, they experience a considerable increase in scholastic workload and professional duties, coupled by frequent feelings of anxiety and uncertainty among their high achieving cohorts and imposter syndrome [51,52]. The struggle of adjusting to a new environment continues in clerkship, as medical students rotate through different hospitals and specialties with frequently changing preceptors, unique medical knowledge base and skill set, and added clinical responsibilities, all on top of continuously striving to academically and clinically excel in each rotation [52]. Findings from the current study demonstrate the consequences of such stressors, as sleeping difficulty (OR 3.72 [1.12–12.35]), experience of being humiliated or ragged by others (OR 2.71 [1.34–5.47]), and financial burden (OR 1.48 [1.06–2.07]) were significantly associated with SI among medical students. Likewise, medical students in clerkship years (OR 1.71 [1.33–2.18]), facing academic difficulty (OR 2.23 [1.39–3.58]), or thinking of dropping out of medical school (OR 3.01 [2.03–4.47]) were at increased risk of SI. Evidently, suicidal behaviour among medical students is a complex and multifactorial phenomenon involving background, personal, and environmental factors that likely converge to produce collinear effects with other highly correlated risk factors.

This study also demonstrated clear deficiency in the number of studies reporting evidence on SA among medical students. This study only identified 3 studies that reported data on SA risk factors, and all three were published within the past two years. While SI often does precede SA [53], most people with suicide ideation do not attempt suicide, and risk factors for suicidal ideation differ from those for suicide attempt [54,55]. In fact, among the four potential SA risk factors assessed in our meta-analysis, only depression (OR 10.34 [7.82–13.67]) was consistent with the results for SI risk, and the remaining three risk factors (alcohol use, female gender, and stress) showed differing effects. Moving forward, the next generation of research on suicidal behaviour among medical students must attend to the important differences between SI and SA, beyond identifying an array of other putative predictors of SA to address the current gap in the literature.

Recognizing the importance of medical student wellbeing, institutional bodies including medical schools have responded with various systemic curricular changes and support programs. However, most programs implemented to date are either post-hoc, therapy-based interventions that occur after-the-fact, or wellness and resilience curricula that lack comprehensive and longitudinal evaluation of their impact on students’ mental health and wellbeing during medical school [9,56]. While these strategies reflect a promising trend in medical education, they tend to focus on teaching self-care to medical students, when the main problem often originates from the environment, not the students [57]. Curricular reform efforts, such as implementation of pass-fail grading system, reduction of preclinical and clinical contact hours, integration of mindfulness, resilience, and stress management curricula, increasing access to mental health counsellors, faculty advisor/mentor programs, and educating faculty about medical student distress have previously shown some effect in improving emotional wellbeing of medical students [58,59]. These strategies may provide useful guidance for fostering medical student wellbeing.

### Limitations

Findings of the current meta-analysis must be interpreted in light of several limitations. Firstly, all included studies relied on self-reported data, and thus are inherently prone to recall bias. Publication bias also could not be excluded. While the funnel-plot analysis of the effect estimates for gender did not demonstrate obvious asymmetry, due to the limited number of studies reporting other outcomes, robust statistical analysis could not be performed to conclusively substantiate the presence of publication bias for the remaining risk factors pooled in this meta-analysis. Third, considering that cross-sectional design was used in almost all included studies, we cannot determine whether these risk factors truly precede SI and SA, thereby representing true risk factors for medical student suicide behaviour. Adding onto the results from this meta-analysis, future efforts should focus on understanding the etiology of the identified risk factors (e.g., depression, stress) to develop practical and effective suicide prevention programs. More research into the temporal relationship between these risk factors and SI and SA among medical students may also be useful to elucidate their true impact. Fourth, in most studies, SI and SA risk factors were only assessed categorically as dichotomous variables. Many of these risk factors, especially those related to mental health (e.g., stress, anxiety), exist on a continuum and such dichotomous representation of these variables as being present or absent is–while understandably difficult to circumvent–an over-simplification of the complex interplay between these risk factors and suicidal behaviour. Therefore, future studies should also investigate the impact of SI and SA at varying levels or intensity for continuous risk factors. Furthermore, future studies investigating the association between varying levels of continuous risk factors and the frequency of SI and SA among medical students would provide greater clarity to the true impact of these risk factors. Finally, several pooled effect estimates were considerably heterogeneous, suggesting significant between-study variability. The reasons for such heterogeneity are not clear and may be effects of differences in population characteristics, selection criteria, and adjustments made to reported odds ratio in the studies. It should also be noted that the current review pooled studies without any geographical restrictions. It therefore follows that any cultural, custom, institutional differences in medical education in the different countries cited in this paper may altogether confound the results and produce between-study heterogeneity as observed above. In view of the considerable heterogeneity across the studies, the aggregate findings reported in this paper need to be treated with caution.

## Supporting information

S1 Fig Forest plots for suicidal ideation risk factors. (PDF)

S2 Fig Forest plots for suicide attempt risk factors. (PDF)

S3 Fig Funnel plot of studies reporting female gender as a SI risk factor. (PDF)

S1 Table PRISMA checklist. (PDF)

S2 Table Search strategy. (PDF)

S1 File (PDF)
