# Supplementary material for: Risk factors for suicidal ideation and suicide attempt among medical students: A meta-analysis
Source: PLoS One. 2021 Dec 22;16(12):e0261785. doi: 10.1371/journal.pone.0261785 (PMC8694469; doi:10.1371/journal.pone.0261785)

# S1 Fig. Forest plots for suicidal ideation risk factors.

## a) Academic difficulty

| Study or Subgroup | log[Odds Ratio] | SE | Weight | Odds Ratio IV, Random, 95% CI |
|---|---|---|---|---|
| Torres et al., 2018 | 0.3772 | 0.3583 | 27.3% | 1.46 [0.72, 2.94] |
| Madadin et al., 2020 | 0.6721 | 0.2692 | 37.0% | 1.96 [1.16, 3.32] |
| Pham et al., 2019 | 0.6981 | 0.576 | 14.0% | 2.01 [0.65, 6.22] |
| Desai et al., 2021 | 1.1939 | 0.6092 | 12.8% | 3.30 [1.00, 10.89] |
| Menezes et al., 2012 | 2.2591 | 0.7561 | 8.9% | 9.57 [2.18, 42.14] |
| **Total (95% CI)** | | | **100.0%** | **2.23 [1.39, 3.58]** |

Heterogeneity: Tau² = 0.09; Chi² = 5.69, df = 4 (P = 0.22); I² = 30%
Test for overall effect: Z = 3.32 (P = 0.0009)

Odds Ratio
IV, Random, 95% CI

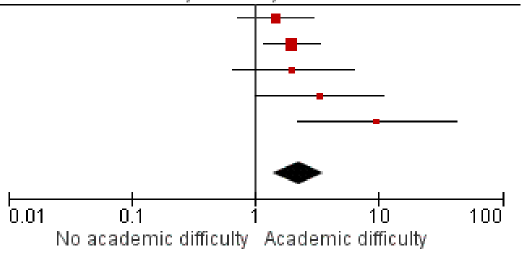


## b) Alcohol use

| Study or Subgroup | log[Odds Ratio] | SE | Weight | Odds Ratio IV, Random, 95% CI |
|---|---|---|---|---|
| Torres et al., 2018 | -0.4102 | 0.3939 | 11.6% | 0.66 [0.31, 1.44] |
| Pham et al., 2019 | 0.0862 | 0.3679 | 12.6% | 1.09 [0.53, 2.24] |
| Jackson et al., 2016 | 0.1656 | 0.1084 | 27.5% | 1.18 [0.95, 1.46] |
| Desalegn et al., 2020 | 0.4511 | 0.3702 | 12.5% | 1.57 [0.76, 3.24] |
| Menezes et al., 2012 | 0.4818 | 0.4739 | 9.0% | 1.62 [0.64, 4.10] |
| Asfaw et al., 2020 | 0.8154 | 0.2264 | 19.9% | 2.26 [1.45, 3.52] |
| Desai et al., 2021 | 1.1632 | 0.5637 | 7.0% | 3.20 [1.06, 9.66] |
| **Total (95% CI)** | | | **100.0%** | **1.42 [1.02, 1.98]** |

Heterogeneity: Tau² = 0.09; Chi² = 12.90, df = 6 (P = 0.04); I² = 54%
Test for overall effect: Z = 2.08 (P = 0.04)

Odds Ratio
IV, Random, 95% CI

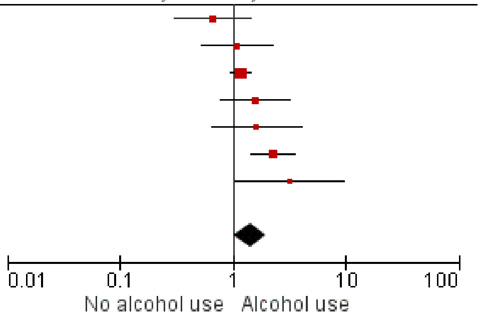


## c) Anxiety

| Study or Subgroup | log[Odds Ratio] | SE | Weight | Odds Ratio IV, Random, 95% CI |
|---|---|---|---|---|
| Asfaw et al., 2020 | 1.0986 | 0.2384 | 70.3% | 3.00 [1.88, 4.79] |
| Torres et al., 2018 | 1.1221 | 0.3669 | 29.7% | 3.07 [1.50, 6.30] |
| **Total (95% CI)** | | | **100.0%** | **3.02 [2.04, 4.47]** |

Heterogeneity: Tau² = 0.00; Chi² = 0.00, df = 1 (P = 0.96); I² = 0%
Test for overall effect: Z = 5.53 (P < 0.00001)

Odds Ratio
IV, Random, 95% CI

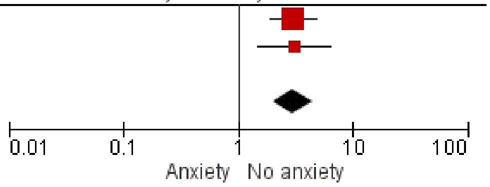


## d) Burnout

| Study or Subgroup | log[Odds Ratio] | SE | Weight | Odds Ratio IV, Random, 95% CI |
|---|---|---|---|---|
| Dyrbye et al., 2008 | 1.4071 | 0.1571 | 63.6% | 4.08 [3.00, 5.56] |
| Talih et al., 2018 | 2.5951 | 0.6392 | 36.4% | 13.40 [3.83, 46.89] |
| **Total (95% CI)** | | | **100.0%** | **6.29 [2.05, 19.30]** |

Heterogeneity: Tau² = 0.49; Chi² = 3.26, df = 1 (P = 0.07); I² = 69%
Test for overall effect: Z = 3.22 (P = 0.001)

Odds Ratio
IV, Random, 95% CI

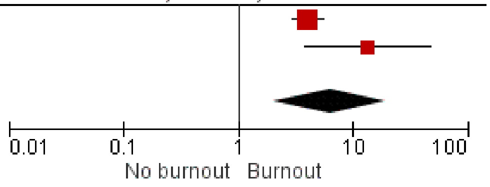

e) Cigarette use

| Study or Subgroup | log[Odds Ratio] | SE | Weight | Odds Ratio IV, Random, 95% CI |
|---|---|---|---|---|
| Desalegn et al., 2020 | -0.6349 | 0.717 | 16.6% | 0.53 [0.13, 2.16] |
| Desai et al., 2021 | -0.1637 | 0.7563 | 15.5% | 0.85 [0.19, 3.74] |
| Menezes et al., 2012 | 0.9927 | 0.6188 | 19.9% | 2.70 [0.80, 9.08] |
| Osama et al., 2014 | 1.1578 | 0.389 | 30.4% | 3.18 [1.48, 6.82] |
| Okasha et al., 1981 | 1.3218 | 0.6834 | 17.7% | 3.75 [0.98, 14.31] |
| **Total (95% CI)** | | | **100.0%** | **1.92 [0.94, 3.92]** |

Heterogeneity: Tau² = 0.29; Chi² = 7.14, df = 4 (P = 0.13); I² = 44%
Test for overall effect: Z = 1.79 (P = 0.07)

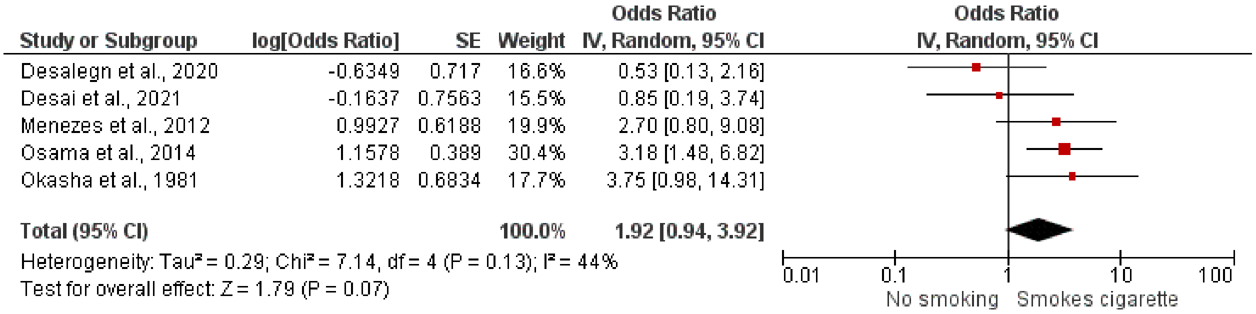


f) Clerkship

| Study or Subgroup | log[Odds Ratio] | SE | Weight | Odds Ratio IV, Random, 95% CI |
|---|---|---|---|---|
| Goebert et al., 2009 | 0.0834 | 0.2468 | 13.7% | 1.09 [0.67, 1.76] |
| Desai et al., 2021 | 0.1823 | 0.4377 | 6.4% | 1.20 [0.51, 2.83] |
| Dyrbye et al., 2008 | 0.3432 | 0.136 | 21.7% | 1.41 [1.08, 1.84] |
| Maser et al., 2019 | 0.4383 | 0.1349 | 21.8% | 1.55 [1.19, 2.02] |
| Tang et al., 2020 | 0.6626 | 0.197 | 17.0% | 1.94 [1.32, 2.85] |
| Ahmed et al., 2016 | 0.9527 | 0.3229 | 9.9% | 2.59 [1.38, 4.88] |
| Menezes et al., 2012 | 1.1314 | 0.5015 | 5.1% | 3.10 [1.16, 8.28] |
| Schwenk et al., 2010 | 1.7712 | 0.5604 | 4.3% | 5.88 [1.96, 17.63] |
| **Total (95% CI)** | | | **100.0%** | **1.71 [1.33, 2.18]** |

Heterogeneity: Tau² = 0.05; Chi² = 14.13, df = 7 (P = 0.05); I² = 50%
Test for overall effect: Z = 4.26 (P < 0.0001)

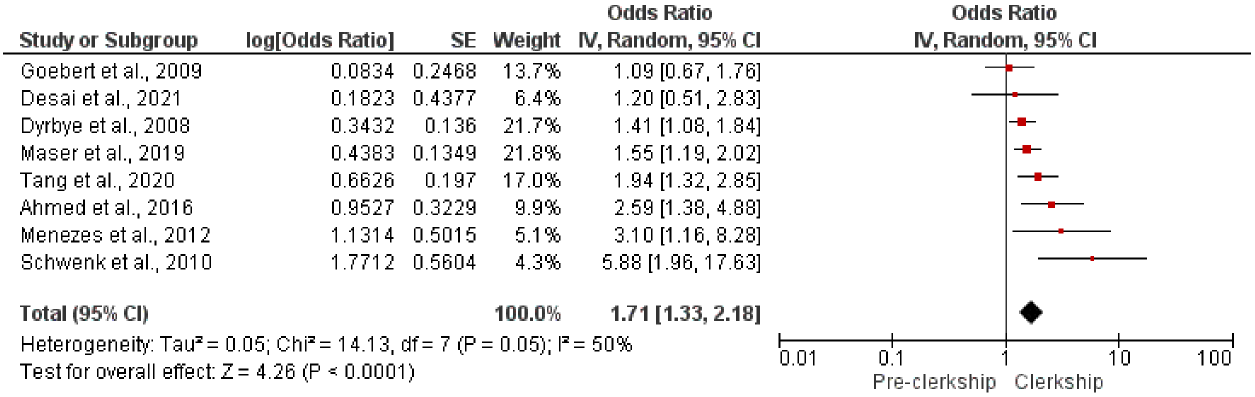


g) Comorbid mental illness

| Study or Subgroup | log[Odds Ratio] | SE | Weight | Odds Ratio IV, Random, 95% CI |
|---|---|---|---|---|
| Desai et al., 2021 | 0.5306 | 0.6347 | 12.9% | 1.70 [0.49, 5.90] |
| Desalegn et al., 2020 | 0.8755 | 0.7335 | 10.9% | 2.40 [0.57, 10.11] |
| Nesan et al., 2020 | 1.3078 | 0.6314 | 13.0% | 3.70 [1.07, 12.75] |
| Osama et al., 2014 | 1.4221 | 0.4068 | 19.4% | 4.15 [1.87, 9.20] |
| Dyrbye et al., 2012 | 2.2394 | 0.2093 | 26.2% | 9.39 [6.23, 14.15] |
| Torres et al., 2018 | 2.4343 | 0.4654 | 17.5% | 11.41 [4.58, 28.40] |
| **Total (95% CI)** | | | **100.0%** | **5.08 [2.81, 9.18]** |

Heterogeneity: Tau² = 0.31; Chi² = 12.81, df = 5 (P = 0.03); I² = 61%
Test for overall effect: Z = 5.37 (P < 0.00001)

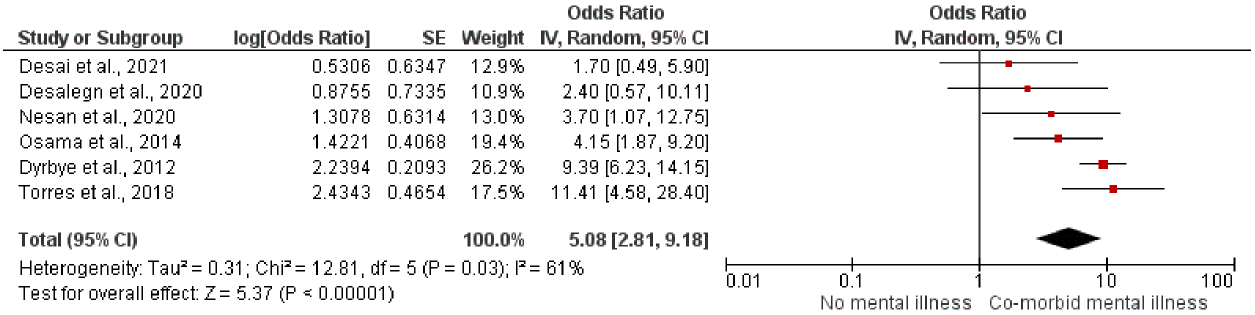


h) Demanding parents

| Study or Subgroup | log[Odds Ratio] | SE | Weight | Odds Ratio IV, Random, 95% CI |
|---|---|---|---|---|
| Ghazanfar et al., 2015 | 0.561 | 0.2727 | 36.4% | 1.75 [1.03, 2.99] |
| Nesan et al., 2020 | 0.5973 | 0.4262 | 14.9% | 1.82 [0.79, 4.19] |
| Osama et al., 2014 | 0.8599 | 0.2356 | 48.7% | 2.36 [1.49, 3.75] |
| **Total (95% CI)** | | | **100.0%** | **2.04 [1.48, 2.81]** |

Heterogeneity: Tau² = 0.00; Chi² = 0.77, df = 2 (P = 0.68); I² = 0%
Test for overall effect: Z = 4.33 (P < 0.0001)

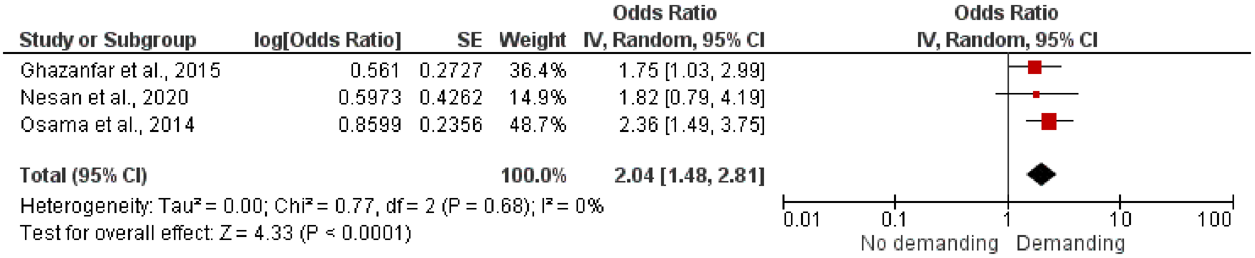

## i) Depression

| Study or Subgroup | log[Odds Ratio] | SE | Weight | Odds Ratio IV, Random, 95% CI |
|---|---|---|---|---|
| Asfaw et al., 2020 | 1.2754 | 0.2415 | 14.8% | 3.58 [2.23, 5.75] |
| Schwenk et al., 2010 | 1.527 | 0.4542 | 8.9% | 4.60 [1.89, 11.21] |
| Ghazanfar et al., 2015 | 1.5614 | 0.2458 | 14.6% | 4.77 [2.94, 7.71] |
| Dyrbye et al., 2008 | 1.8727 | 0.174 | 16.9% | 6.51 [4.63, 9.15] |
| Torres et al., 2018 | 1.8886 | 0.4493 | 9.0% | 6.61 [2.74, 15.95] |
| Sobowale et al., 2014 | 2.1366 | 0.4321 | 9.4% | 8.47 [3.63, 19.76] |
| Desalegn et al., 2020 | 2.3145 | 0.2029 | 16.0% | 10.12 [6.80, 15.06] |
| Pham et al., 2019 | 3.038 | 0.3889 | 10.5% | 20.86 [9.74, 44.71] |
| **Total (95% CI)** | | | **100.0%** | **6.87 [4.80, 9.82]** |

Heterogeneity: $Tau^2 = 0.17$; $Chi^2 = 22.32$, df = 7 (P = 0.002); $I^2 = 69\%$
Test for overall effect: Z = 10.57 (P < 0.00001)

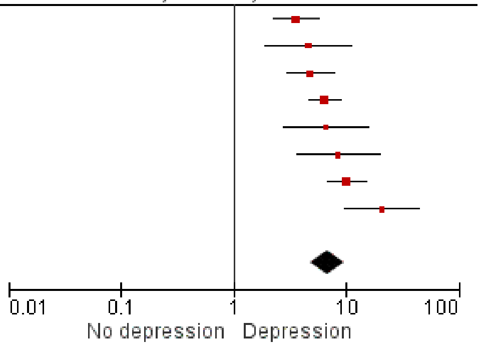


## j) Fatigue

| Study or Subgroup | log[Odds Ratio] | SE | Weight | Odds Ratio IV, Random, 95% CI |
|---|---|---|---|---|
| Ghazanfar et al., 2015 | 0.7638 | 0.2383 | 53.5% | 2.15 [1.35, 3.42] |
| Tang et al., 2020 | 1.8099 | 0.3646 | 46.5% | 6.11 [2.99, 12.48] |
| **Total (95% CI)** | | | **100.0%** | **3.49 [1.26, 9.71]** |

Heterogeneity: $Tau^2 = 0.45$; $Chi^2 = 5.77$, df = 1 (P = 0.02); $I^2 = 83\%$
Test for overall effect: Z = 2.40 (P = 0.02)

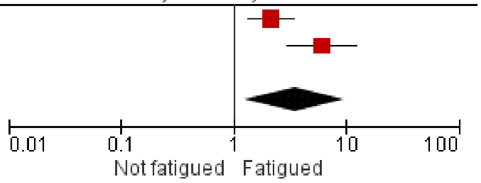


## k) Female

| Study or Subgroup | log[Odds Ratio] | SE | Weight | Odds Ratio IV, Random, 95% CI |
|---|---|---|---|---|
| Menezes et al., 2012 | -0.429 | 0.4672 | 4.3% | 0.65 [0.26, 1.63] |
| Pham et al., 2019 | -0.3857 | 0.4527 | 4.4% | 0.68 [0.28, 1.65] |
| Compton et al., 2008 | -0.2107 | 0.2777 | 6.3% | 0.81 [0.47, 1.40] |
| Torres et al., 2018 | -0.0149 | 0.3617 | 5.3% | 0.99 [0.48, 2.00] |
| Tang et al., 2020 | 0.0486 | 0.1964 | 7.3% | 1.05 [0.71, 1.54] |
| Tareq et al., 2020 | 0.086 | 0.2893 | 6.2% | 1.09 [0.62, 1.92] |
| Dyrbye et al., 2008 | 0.1205 | 0.1348 | 7.9% | 1.13 [0.87, 1.47] |
| Maser et al., 2019 | 0.1989 | 0.1276 | 8.0% | 1.22 [0.95, 1.57] |
| Asfaw et al., 2020 | 0.27 | 0.2207 | 7.0% | 1.31 [0.85, 2.02] |
| Osama et al., 2014 | 0.3635 | 0.2372 | 6.8% | 1.44 [0.90, 2.29] |
| Schwenk et al., 2010 | 0.4406 | 0.4669 | 4.3% | 1.55 [0.62, 3.88] |
| Madadin et al., 2020 | 0.4952 | 0.2573 | 6.6% | 1.64 [0.99, 2.72] |
| Ahmed et al., 2016 | 0.4955 | 0.2745 | 6.4% | 1.64 [0.96, 2.81] |
| Yousaf et al., 2016 | 0.8214 | 0.4502 | 4.4% | 2.27 [0.94, 5.49] |
| Khosravi & Kasaeiyan, 2020 | 1.4026 | 0.3163 | 5.9% | 4.07 [2.19, 7.56] |
| Desalegn et al., 2020 | 1.6506 | 0.2148 | 7.1% | 5.21 [3.42, 7.94] |
| Desai et al., 2021 | 2.1861 | 0.8756 | 1.9% | 8.90 [1.60, 49.51] |
| **Total (95% CI)** | | | **100.0%** | **1.47 [1.13, 1.92]** |

Heterogeneity: $Tau^2 = 0.21$; $Chi^2 = 71.96$, df = 16 (P < 0.00001); $I^2 = 78\%$
Test for overall effect: Z = 2.86 (P = 0.004)

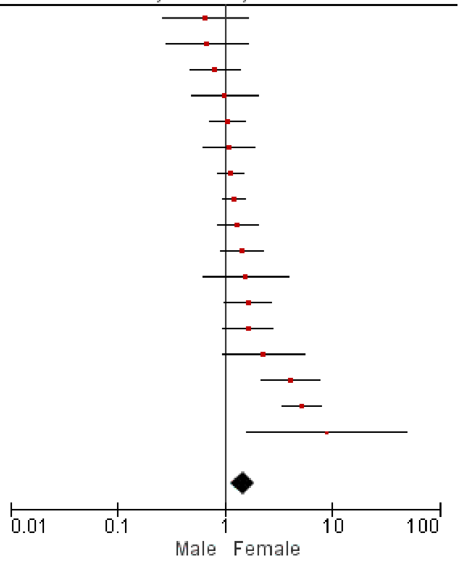

## l) Family history of mental illness

| Study or Subgroup | log[Odds Ratio] | SE | Weight | Odds Ratio IV, Random, 95% CI |
|---|---|---|---|---|
| Asfaw et al., 2020 | -0.0943 | 0.3825 | 31.7% | 0.91 [0.43, 1.93] |
| Desalegn et al., 2020 | 0.6313 | 0.4359 | 28.9% | 1.88 [0.80, 4.42] |
| Ghazanfar et al., 2015 | 1.0935 | 0.2441 | 39.4% | 2.98 [1.85, 4.82] |
| **Total (95% CI)** | | | **100.0%** | **1.79 [0.86, 3.74]** |

Heterogeneity: Tau² = 0.30; Chi² = 6.91, df = 2 (P = 0.03); I² = 71%
Test for overall effect: Z = 1.56 (P = 0.12)

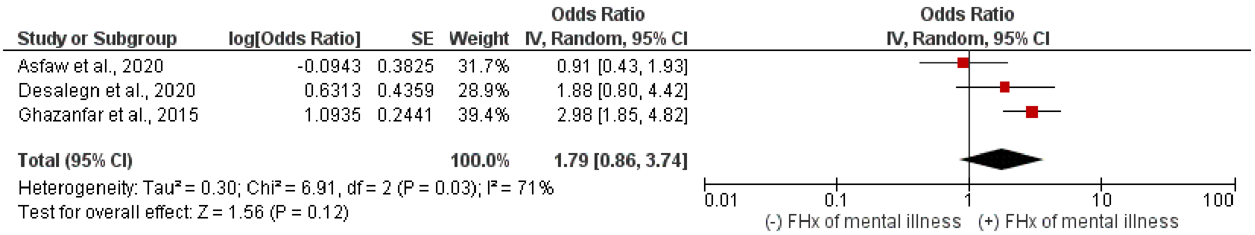


## m) Family history of suicide attempt

| Study or Subgroup | log[Odds Ratio] | SE | Weight | Odds Ratio IV, Random, 95% CI |
|---|---|---|---|---|
| Asfaw et al., 2020 | 0.3436 | 0.4192 | 69.9% | 1.41 [0.62, 3.21] |
| Desai et al., 2021 | -0.3567 | 0.6392 | 30.1% | 0.70 [0.20, 2.45] |
| **Total (95% CI)** | | | **100.0%** | **1.14 [0.57, 2.27]** |

Heterogeneity: Tau² = 0.00; Chi² = 0.84, df = 1 (P = 0.36); I² = 0%
Test for overall effect: Z = 0.38 (P = 0.70)

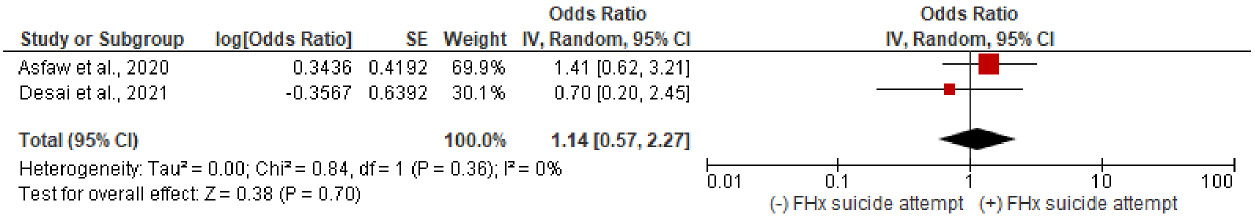


## n) Financial burden

| Study or Subgroup | log[Odds Ratio] | SE | Weight | Odds Ratio IV, Random, 95% CI |
|---|---|---|---|---|
| Tang et al., 2020 | -0.0225 | 0.1935 | 29.1% | 0.98 [0.67, 1.43] |
| Dyrbye et al., 2008 | 0.3865 | 0.1671 | 32.1% | 1.47 [1.06, 2.04] |
| Pham et al., 2019 | 0.6471 | 0.2544 | 22.9% | 1.91 [1.16, 3.14] |
| Ghazanfar et al., 2015 | 0.7927 | 0.3473 | 15.9% | 2.21 [1.12, 4.36] |
| **Total (95% CI)** | | | **100.0%** | **1.48 [1.06, 2.07]** |

Heterogeneity: Tau² = 0.06; Chi² = 6.74, df = 3 (P = 0.08); I² = 55%
Test for overall effect: Z = 2.29 (P = 0.02)

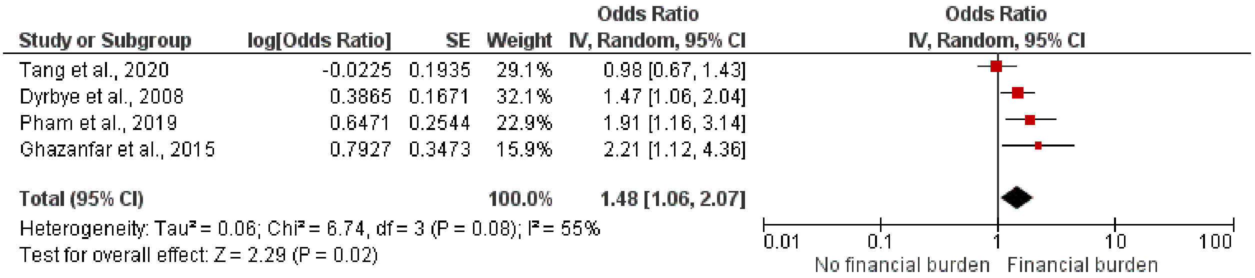


## o) History of physical or sexual abuse

| Study or Subgroup | log[Odds Ratio] | SE | Weight | Odds Ratio IV, Random, 95% CI |
|---|---|---|---|---|
| Madadin et al., 2020 | 0.7602 | 0.2815 | 65.9% | 2.14 [1.23, 3.71] |
| Menezes et al., 2012 | 1.2408 | 0.5392 | 18.0% | 3.46 [1.20, 9.95] |
| Desai et al., 2021 | 1.361 | 0.5684 | 16.2% | 3.90 [1.28, 11.88] |
| **Total (95% CI)** | | | **100.0%** | **2.57 [1.64, 4.02]** |

Heterogeneity: Tau² = 0.00; Chi² = 1.27, df = 2 (P = 0.53); I² = 0%
Test for overall effect: Z = 4.13 (P < 0.0001)

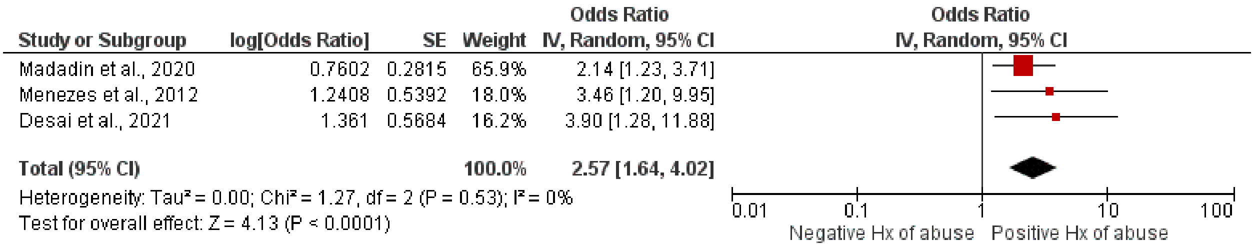


## p) History of physical assault

| Study or Subgroup | log[Odds Ratio] | SE | Weight | Odds Ratio IV, Random, 95% CI |
|---|---|---|---|---|
| Nesan et al., 2020 | 1.0355 | 0.4697 | 45.6% | 2.82 [1.12, 7.07] |
| Osama et al., 2014 | 1.3242 | 0.4298 | 54.4% | 3.76 [1.62, 8.73] |
| **Total (95% CI)** | | | **100.0%** | **3.30 [1.77, 6.14]** |

Heterogeneity: Tau² = 0.00; Chi² = 0.21, df = 1 (P = 0.65); I² = 0%
Test for overall effect: Z = 3.76 (P = 0.0002)

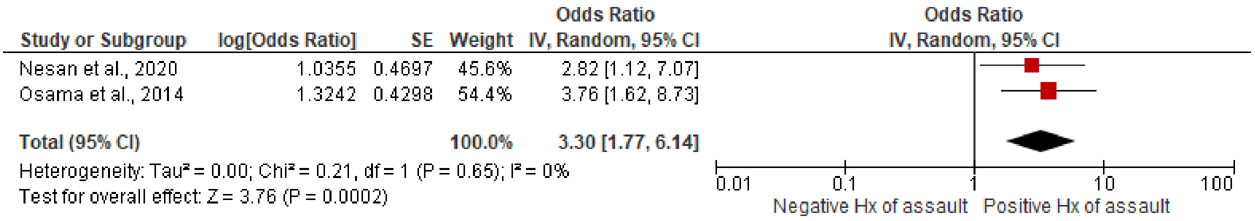

## q) Living alone

| Study or Subgroup | log[Odds Ratio] | SE | Weight | Odds Ratio IV, Random, 95% CI |
|---|---|---|---|---|
| Pham et al., 2019 | -0.2485 | 0.8082 | 8.3% | 0.78 [0.16, 3.80] |
| Nesan et al., 2020 | 0.849 | 0.301 | 59.6% | 2.34 [1.30, 4.22] |
| Torres et al., 2018 | 0.8713 | 0.41 | 32.1% | 2.39 [1.07, 5.34] |
| **Total (95% CI)** | | | **100.0%** | **2.15 [1.36, 3.39]** |

Heterogeneity: Tau² = 0.00; Chi² = 1.72, df = 2 (P = 0.42); I² = 0%
Test for overall effect: Z = 3.29 (P = 0.0010)

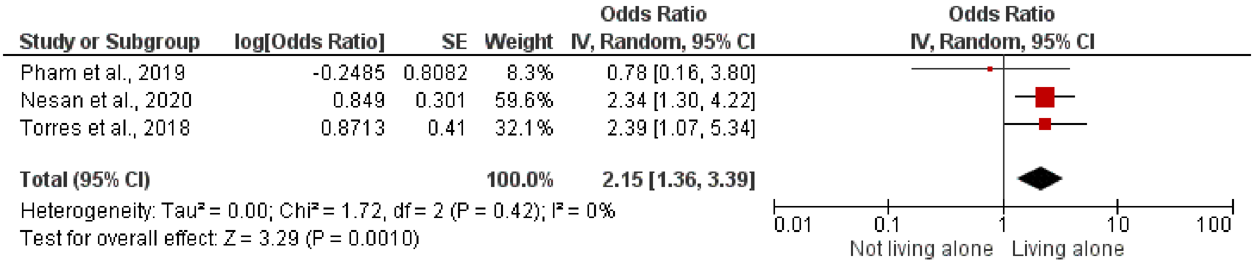


## r) Living away from home

| Study or Subgroup | log[Odds Ratio] | SE | Weight | Odds Ratio IV, Random, 95% CI |
|---|---|---|---|---|
| Pham et al., 2019 | -0.1374 | 0.4155 | 20.0% | 0.87 [0.39, 1.97] |
| Nesan et al., 2020 | 0.849 | 0.301 | 20.8% | 2.34 [1.30, 4.22] |
| Desai et al., 2021 | 0.8755 | 0.6287 | 18.2% | 2.40 [0.70, 8.23] |
| Torres et al., 2018 | 1.2242 | 0.3673 | 20.4% | 3.40 [1.66, 6.99] |
| Ahmed et al., 2016 | 3.6207 | 0.3355 | 20.6% | 37.36 [19.36, 72.12] |
| **Total (95% CI)** | | | **100.0%** | **3.68 [0.98, 13.84]** |

Heterogeneity: Tau² = 2.11; Chi² = 61.65, df = 4 (P < 0.00001); I² = 94%
Test for overall effect: Z = 1.93 (P = 0.05)

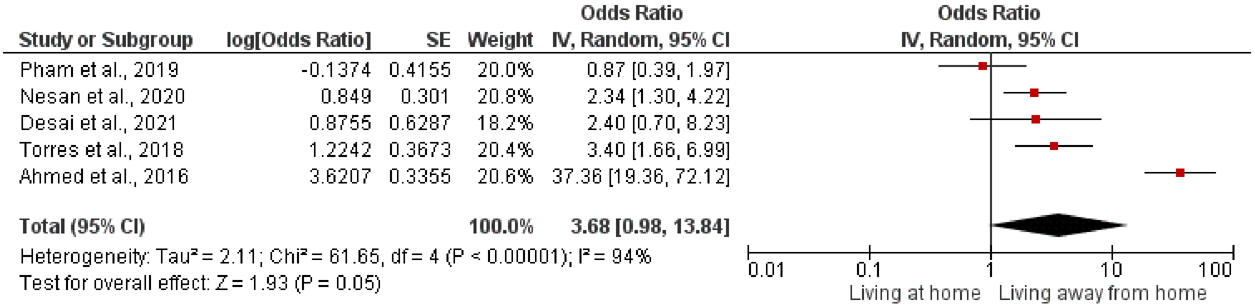


## s) Parental neglect

| Study or Subgroup | log[Odds Ratio] | SE | Weight | Odds Ratio IV, Random, 95% CI |
|---|---|---|---|---|
| Madadin et al., 2020 | -0.0564 | 0.3383 | 30.1% | 0.95 [0.49, 1.83] |
| Menezes et al., 2012 | 1.2513 | 0.6342 | 20.6% | 3.49 [1.01, 12.11] |
| Osama et al., 2014 | 1.3638 | 0.3667 | 29.1% | 3.91 [1.91, 8.02] |
| Nesan et al., 2020 | 1.4441 | 0.6454 | 20.3% | 4.24 [1.20, 15.02] |
| **Total (95% CI)** | | | **100.0%** | **2.53 [1.08, 5.92]** |

Heterogeneity: Tau² = 0.51; Chi² = 10.26, df = 3 (P = 0.02); I² = 71%
Test for overall effect: Z = 2.15 (P = 0.03)

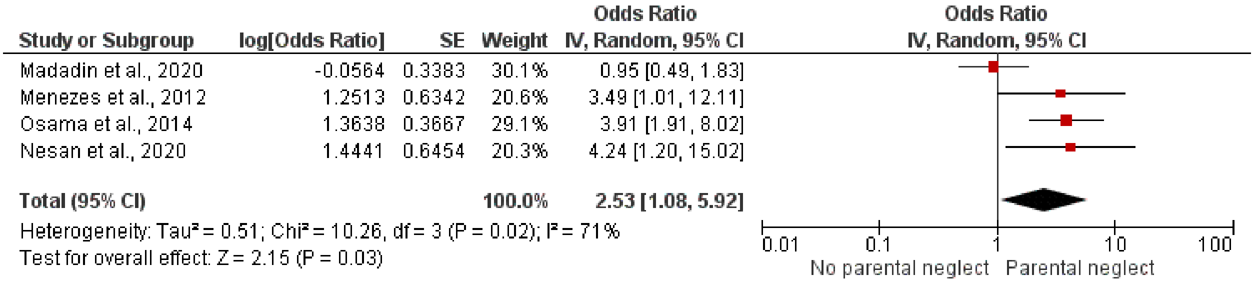


## t) Poor social support

| Study or Subgroup | log[Odds Ratio] | SE | Weight | Odds Ratio IV, Random, 95% CI |
|---|---|---|---|---|
| Desalegn et al., 2020 | 0.6678 | 0.5451 | 17.3% | 1.95 [0.67, 5.68] |
| Asfaw et al., 2020 | 0.9439 | 0.3063 | 32.4% | 2.57 [1.41, 4.68] |
| Torres et al., 2018 | 0.9567 | 0.3606 | 28.0% | 2.60 [1.28, 5.28] |
| Ghazanfar et al., 2015 | 2.0606 | 0.4467 | 22.3% | 7.85 [3.27, 18.84] |
| **Total (95% CI)** | | | **100.0%** | **3.15 [1.84, 5.41]** |

Heterogeneity: Tau² = 0.14; Chi² = 5.66, df = 3 (P = 0.13); I² = 47%
Test for overall effect: Z = 4.17 (P < 0.0001)

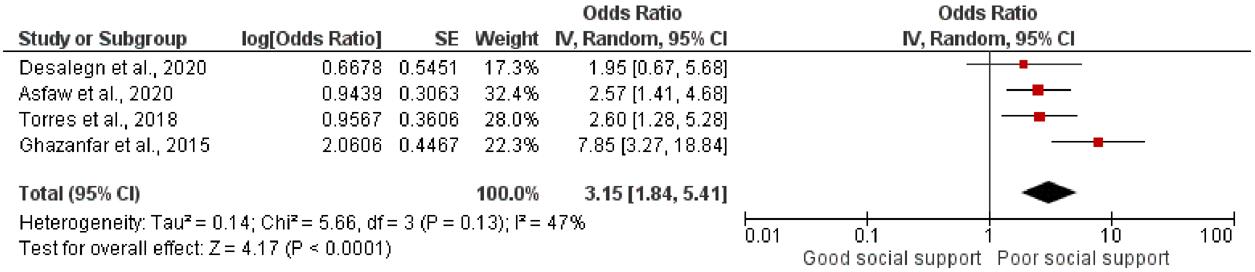


## u) Ragged by others

| Study or Subgroup | log[Odds Ratio] | SE | Weight | Odds Ratio IV, Random, 95% CI |
|---|---|---|---|---|
| Osama et al., 2014 | 0.9163 | 0.4378 | 67.3% | 2.50 [1.06, 5.90] |
| Menezes et al., 2012 | 1.1585 | 0.6283 | 32.7% | 3.19 [0.93, 10.91] |
| **Total (95% CI)** | | | **100.0%** | **2.71 [1.34, 5.47]** |

Heterogeneity: Tau² = 0.00; Chi² = 0.10, df = 1 (P = 0.75); I² = 0%
Test for overall effect: Z = 2.77 (P = 0.006)

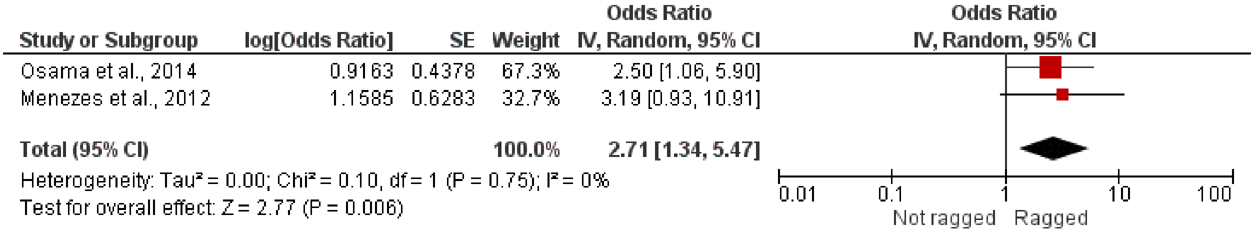

## v) Sleep difficulty

| Study or Subgroup | Difficulty sleeping Events | Difficulty sleeping Total | No sleep difficulty Events | No sleep difficulty Total | Weight | Odds Ratio IV, Random, 95% CI |
|---|---|---|---|---|---|---|
| Ghazanfar et al., 2015 | 15 | 121 | 62 | 1011 | 56.2% | 2.17 [1.19, 3.94] |
| Okasha et al., 1981 | 25 | 30 | 41 | 102 | 43.8% | 7.44 [2.63, 21.02] |
| **Total (95% CI)** | | **151** | | **1113** | **100.0%** | **3.72 [1.12, 12.35]** |
| Total events | 40 | | 103 | | | |

Heterogeneity: Tau² = 0.57; Chi² = 4.07, df = 1 (P = 0.04); I² = 75%
Test for overall effect: Z = 2.15 (P = 0.03)

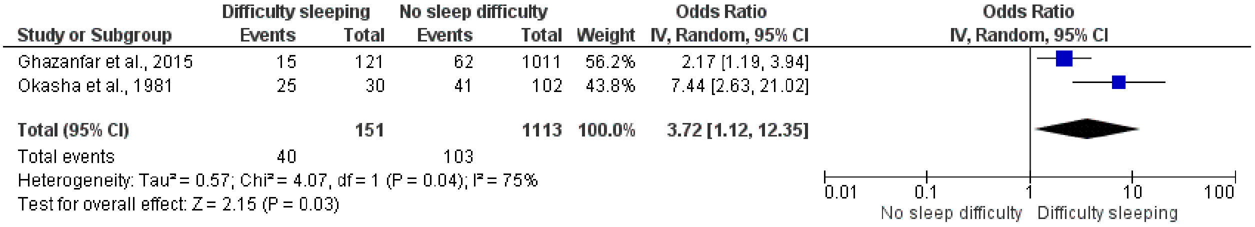


## w) Stress

| Study or Subgroup | log[Odds Ratio] | SE | Weight | Odds Ratio IV, Random, 95% CI |
|---|---|---|---|---|
| Asfaw et al., 2020 | 0.2546 | 0.2128 | 27.2% | 1.29 [0.85, 1.96] |
| Menezes et al., 2012 | 1.1585 | 0.6283 | 19.8% | 3.19 [0.93, 10.91] |
| Compton et al., 2008 | 1.2326 | 0.1865 | 27.5% | 3.43 [2.38, 4.94] |
| Tang et al., 2020 | 2.6519 | 0.3315 | 25.5% | 14.18 [7.41, 27.16] |
| **Total (95% CI)** | | | **100.0%** | **3.72 [1.39, 9.94]** |

Heterogeneity: Tau² = 0.88; Chi² = 38.21, df = 3 (P < 0.00001); I² = 92%
Test for overall effect: Z = 2.62 (P = 0.009)

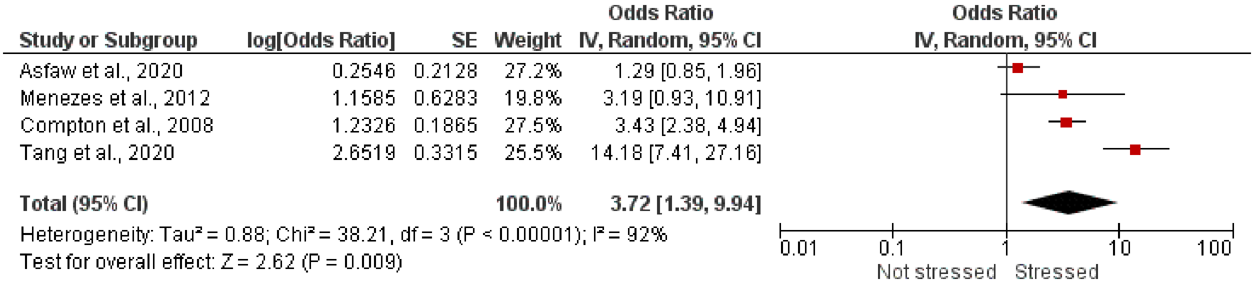


## x) Substance abuse

| Study or Subgroup | log[Odds Ratio] | SE | Weight | Odds Ratio IV, Random, 95% CI |
|---|---|---|---|---|
| Jackson et al., 2016 | 0.1656 | 0.1084 | 34.8% | 1.18 [0.95, 1.46] |
| Desai et al., 2021 | 0.47 | 0.7335 | 25.4% | 1.60 [0.38, 6.74] |
| Menezes et al., 2012 | 2.2083 | 1.0274 | 20.0% | 9.10 [1.21, 68.17] |
| Osama et al., 2014 | 3.3516 | 1.0421 | 19.8% | 28.55 [3.70, 220.10] |
| **Total (95% CI)** | | | **100.0%** | **3.60 [0.91, 14.25]** |

Heterogeneity: Tau² = 1.40; Chi² = 13.14, df = 3 (P = 0.004); I² = 77%
Test for overall effect: Z = 1.83 (P = 0.07)

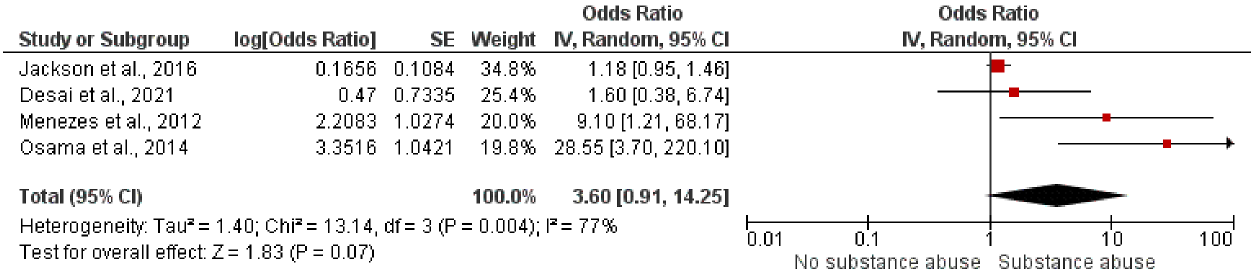


## y) Thoughts of dropping out

| Study or Subgroup | log[Odds Ratio] | SE | Weight | Odds Ratio IV, Random, 95% CI |
|---|---|---|---|---|
| Torres et al., 2018 | 1.0296 | 0.4115 | 23.9% | 2.80 [1.25, 6.27] |
| Ghazanfar et al., 2015 | 1.0913 | 0.2658 | 57.2% | 2.98 [1.77, 5.01] |
| Schwenk et al., 2010 | 1.2321 | 0.4619 | 18.9% | 3.43 [1.39, 8.48] |
| **Total (95% CI)** | | | **100.0%** | **3.01 [2.03, 4.47]** |

Heterogeneity: Tau² = 0.00; Chi² = 0.11, df = 2 (P = 0.95); I² = 0%
Test for overall effect: Z = 5.49 (P < 0.00001)

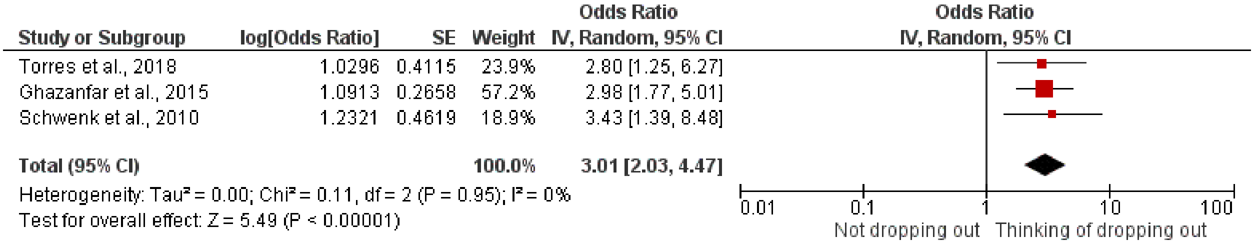

Supplement: S1 Fig — (PDF) [file pone.0261785.s001.pdf]
