# Supplementary figures and images for: Risk factors for suicidal ideation and suicide attempt among medical students: A meta-analysis
Source: PLoS One. 2021 Dec 22;16(12):e0261785. doi: 10.1371/journal.pone.0261785 (PMC8694469; doi:10.1371/journal.pone.0261785)

## S2 Fig. Forest plots for suicide attempt risk factors.

### a) Alcohol use

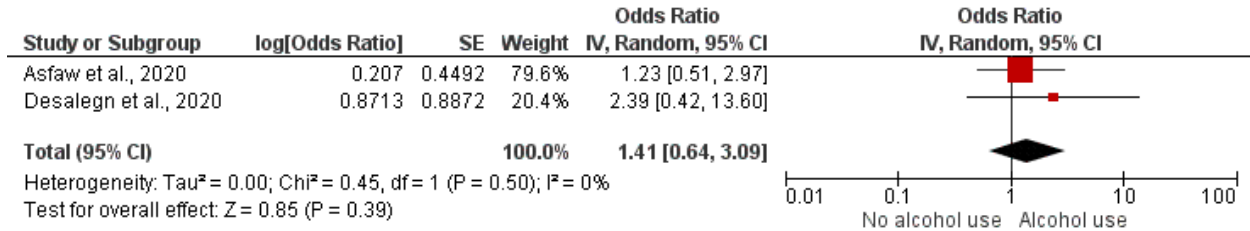

### b) Depression

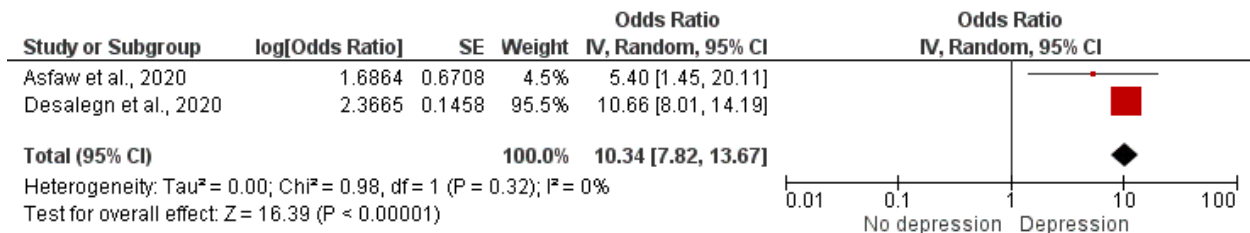

### c) Female

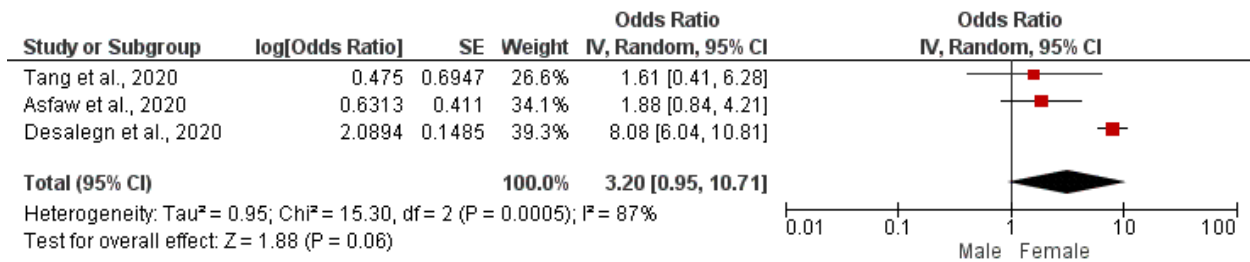

### d) Stress

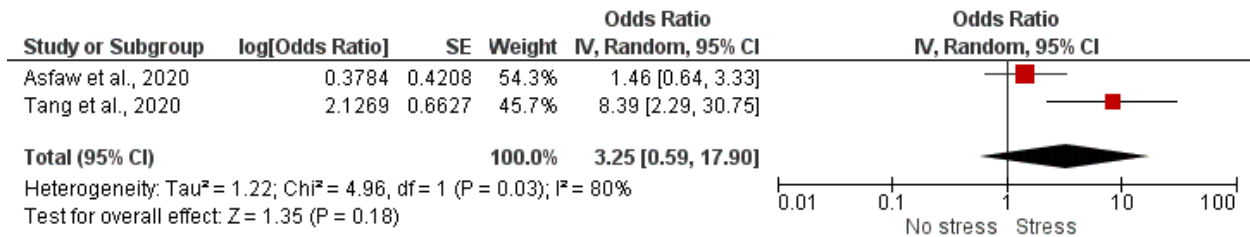

Supplement: S2 Fig — (PDF) [file pone.0261785.s002.pdf]

**S3 Fig. Funnel plot of studies reporting female gender as a  
SI risk factor.**

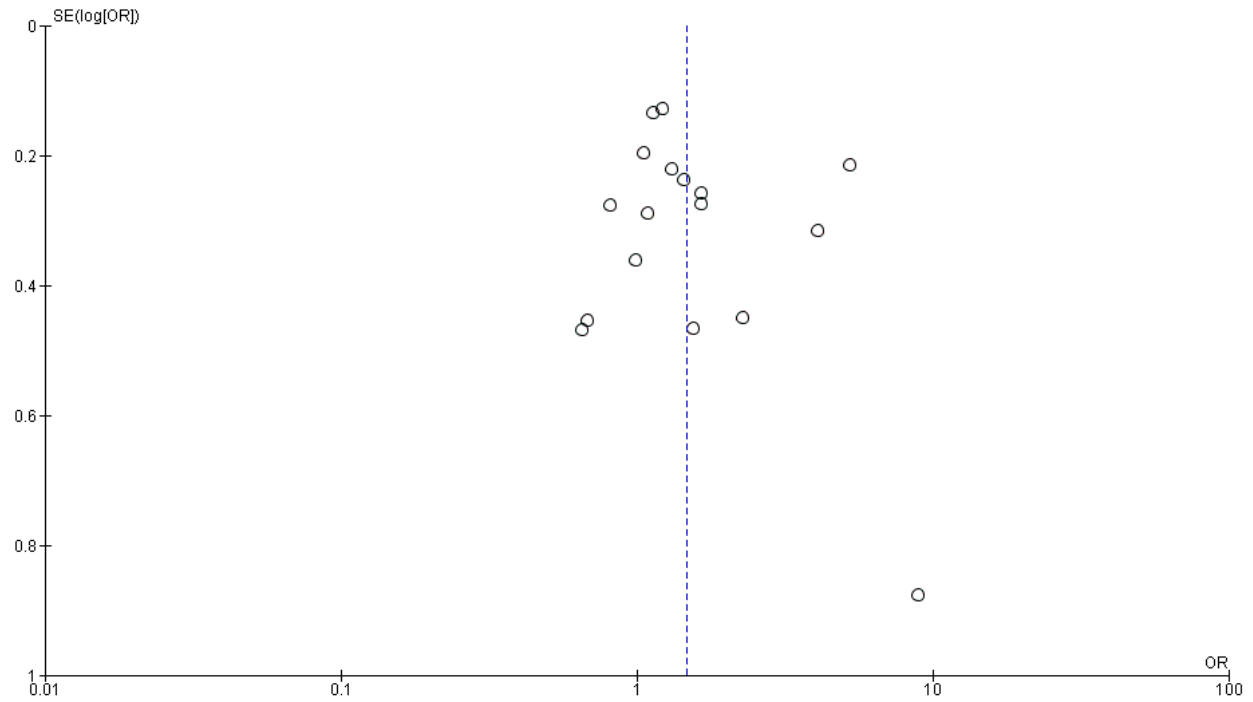

Supplement: S3 Fig — (PDF) [file pone.0261785.s003.pdf]
