# Supplementary material for: Risk factors for suicidal ideation and suicide attempt among medical students: A meta-analysis
Source: PLoS One. 2021 Dec 22;16(12):e0261785. doi: 10.1371/journal.pone.0261785 (PMC8694469; doi:10.1371/journal.pone.0261785)
Supplement: S2 Table — (PDF) [file pone.0261785.s005.pdf]

S2 Table. Search strategy (performed on March 21, 2021).

MEDLINE® ALL (Ovid, 1946 to March 19, 2021)	1. Students, Medical/<br>2. education, medical/ or education, medical, undergraduate/<br>3. ((medical* or medicine*) adj2 (educat* or train* or student*)).ti,ab,kf.<br>4. or/1-3<br>5. suicide/ or suicidal ideation/ or suicide, attempted/ or suicide, completed/<br>6. suicid*.ti,ab,kf.<br>7. or/5-6<br>8. 4 and 7<br>9. case reports/<br>10. (editorial or letter or comments).pt.<br>11. (case report* or case stud*).ti,ab,kf.<br>12. or/9-11<br>13. 8 not 12<br><br>Results: **661** citations retrieved
Embase (Ovid, 1947 to 2021 March 19)	1. medical student/<br>2. medical education/<br>3. ((medical* or medicine*) adj2 (educat* or train* or student*)).ti,ab,kw.<br>4. or/1-3<br>5. suicidal behavior/ or suicidal ideation/ or self immolation/ or self poisoning/ or suicide/ or suicide attempt/<br>6. suicid*.ti,ab,kw.<br>7. or/5-6<br>8. 4 and 7<br>9. case report/ or case study/<br>10. (case report* or case stud*).ti,ab,kw.<br>11. (Letter or Editorial or conference abstract).pt.<br>12. abstract report/ or letter/<br>13. or/9-12<br>14. 8 not 13<br><br>Results: **1,286** citations retrieved
APA PsycInfo (Ovid, 1806 to March Week 3 2021)	1. medical students/<br>2. medical education/<br>3. ((medical* or medicine*) adj2 (educat* or train* or student*)).tw.<br>4. or/1-3<br>5. suicide/ or suicidal ideation/ or attempted suicide/ or suicidality/ or suicidology/<br>6. suicid*.tw.<br>7. or/5-6<br>8. case report/<br>9. (case report* or case stud*).tw.

	10. (Book or Authored Book or Edited Book or Dissertation Abstract or Encyclopedia).pt.<br>11. or/8-10<br>12. 4 and 7<br>13. 12 not 11<br><br>Results: **418** citations retrieved
CINAHL (EBSCOHost, 1976-2021)	((MH "Students, Medical") OR (MH "Education, Medical") OR (TI (((medical* or medicine*) N2 (educat* or train* or student*))) OR AB (((medical* or medicine*) N2 (educat* or train* or student*))))) AND (((MH "Suicide") OR (MH "Suicide, Attempted") OR (MH "Suicidal Ideation")) OR (TI suicid* OR AB suicid*))<br><br>Results: **357** citations retrieved
Education Source (EBSCOHost, 1974-2021)	(((DE "Medical education") OR (DE "Medical students" OR DE "Women medical students")) OR (AB ((medical* or medicine*) N2 (educat* or train* or student*)) OR TI ((medical* or medicine*) N2 (educat* or train* or student*)))) AND ((DE "Suicidal behavior in college students") OR (DE "Suicidal behavior in young adults") OR (AB suicid* OR TI suicid*))<br><br>Results: **85** citations retrieved
Scopus	(TITLE-ABS-KEY ((medical* OR medicine*) W/2 (educat* OR train* OR student*)) AND TITLE-ABS-KEY (suicid*)) AND (LIMIT-TO (SRCTYPE , "j")) AND (LIMIT-TO (DOCTYPE , "ar") OR LIMIT-TO (DOCTYPE , "sh"))<br><br>Results: **1,246** citations retrieved
