# Supplementary material for: Risk factors for suicidal ideation and suicide attempt among medical students: A meta-analysis
Source: PLoS One. 2021 Dec 22;16(12):e0261785. doi: 10.1371/journal.pone.0261785 (PMC8694469; doi:10.1371/journal.pone.0261785)

SUPPLEMENTAL DIGITAL APPENDICES

Table of Contents

S1 Table: PRISMA checklist……………………….…………………………2

S2 Table: Search strategy…………………………..………………………….5

S1 Figure: Forest plots for suicidal ideation risk factors …………..…….7

S2 Figure: Forest plots for suicide attempt risk factors.…………………13

S3 Figure: Funnel plot for publication bias assessment …………………14

S1 Table. PRISMA checklist.

Section/topic	#	Checklist item	Reported on page #
**TITLE**			
Title	1	Identify the report as a systematic review, meta-analysis, or both.	1
**ABSTRACT**			
Structured summary	2	Provide a structured summary including, as applicable: background; objectives; data sources; study eligibility criteria, participants, and interventions; study appraisal and synthesis methods; results; limitations; conclusions and implications of key findings; systematic review registration number.	2
**INTRODUCTION**			
Rationale	3	Describe the rationale for the review in the context of what is already known.	5
Objectives	4	Provide an explicit statement of questions being addressed with reference to participants, interventions, comparisons, outcomes, and study design (PICOS).	5
**METHODS**			
Protocol and registration	5	Indicate if a review protocol exists, if and where it can be accessed (e.g., Web address), and, if available, provide registration information including registration number.	5
Eligibility criteria	6	Specify study characteristics (e.g., PICOS, length of follow-up) and report characteristics (e.g., years considered, language, publication status) used as criteria for eligibility, giving rationale.	6
Information sources	7	Describe all information sources (e.g., databases with dates of coverage, contact with study authors to identify additional studies) in the search and date last searched.	6
Search	8	Present full electronic search strategy for at least one database, including any limits used, such that it could be repeated.	S2 table
Study selection	9	State the process for selecting studies (i.e., screening, eligibility, included in systematic review, and, if applicable, included in the meta-analysis).	7

Data collection process	10	Describe method of data extraction from reports (e.g., piloted forms, independently, in duplicate) and any processes for obtaining and confirming data from investigators.	7
Data items	11	List and define all variables for which data were sought (e.g., PICOS, funding sources) and any assumptions and simplifications made.	7
Risk of bias in individual studies	12	Describe methods used for assessing risk of bias of individual studies (including specification of whether this was done at the study or outcome level), and how this information is to be used in any data synthesis.	7
Summary measures	13	State the principal summary measures (e.g., risk ratio, difference in means).	8
Synthesis of results	14	Describe the methods of handling data and combining results of studies, if done, including measures of consistency (e.g., I^2) for each meta-analysis.	8
Risk of bias across studies	15	Specify any assessment of risk of bias that may affect the cumulative evidence (e.g., publication bias, selective reporting within studies).	7
Additional analyses	16	Describe methods of additional analyses (e.g., sensitivity or subgroup analyses, meta-regression), if done, indicating which were pre-specified.	N/A
**RESULTS**			
Study selection	17	Give numbers of studies screened, assessed for eligibility, and included in the review, with reasons for exclusions at each stage, ideally with a flow diagram.	9
Study characteristics	18	For each study, present characteristics for which data were extracted (e.g., study size, PICOS, follow-up period) and provide the citations.	9
Risk of bias within studies	19	Present data on risk of bias of each study and, if available, any outcome level assessment (see item 12).	19
Results of individual studies	20	For all outcomes considered (benefits or harms), present, for each study: (a) simple summary data for each intervention group (b) effect estimates and confidence intervals, ideally with a forest plot.	S1 figure and S2 figure
Synthesis of results	21	Present results of each meta-analysis done, including confidence intervals and measures of consistency.	13-19
Risk of bias across studies	22	Present results of any assessment of risk of bias across studies (see Item 15).	19

Additional analysis	23	Give results of additional analyses, if done (e.g., sensitivity or subgroup analyses, meta-regression [see Item 16]).	N/A
**DISCUSSION**			
Summary of evidence	24	Summarize the main findings including the strength of evidence for each main outcome; consider their relevance to key groups (e.g., healthcare providers, users, and policy makers).	19
Limitations	25	Discuss limitations at study and outcome level (e.g., risk of bias), and at review-level (e.g., incomplete retrieval of identified research, reporting bias).	23
Conclusions	26	Provide a general interpretation of the results in the context of other evidence, and implications for future research.	22
**FUNDING**			
Funding	27	Describe sources of funding for the systematic review and other support (e.g., supply of data); role of funders for the systematic review.	24

S2 Table. Search strategy (performed on March 21, 2021).

MEDLINE® ALL (Ovid, 1946 to March 19, 2021)	1. Students, Medical/<br>2. education, medical/ or education, medical, undergraduate/<br>3. ((medical* or medicine*) adj2 (educat* or train* or student*)).ti,ab,kf.<br>4. or/1-3<br>5. suicide/ or suicidal ideation/ or suicide, attempted/ or suicide, completed/<br>6. suicid*.ti,ab,kf.<br>7. or/5-6<br>8. 4 and 7<br>9. case reports/<br>10. (editorial or letter or comments).pt.<br>11. (case report* or case stud*).ti,ab,kf.<br>12. or/9-11<br>13. 8 not 12<br><br>Results: **661** citations retrieved
Embase (Ovid, 1947 to 2021 March 19)	1. medical student/<br>2. medical education/<br>3. ((medical* or medicine*) adj2 (educat* or train* or student*)).ti,ab,kw.<br>4. or/1-3<br>5. suicidal behavior/ or suicidal ideation/ or self immolation/ or self poisoning/ or suicide/ or suicide attempt/<br>6. suicid*.ti,ab,kw.<br>7. or/5-6<br>8. 4 and 7<br>9. case report/ or case study/<br>10. (case report* or case stud*).ti,ab,kw.<br>11. (Letter or Editorial or conference abstract).pt.<br>12. abstract report/ or letter/<br>13. or/9-12<br>14. 8 not 13<br><br>Results: **1,286** citations retrieved

APA PsycInfo (Ovid, 1806 to March Week 3 2021)	1. medical students/<br>2. medical education/<br>3. ((medical* or medicine*) adj2 (educat* or train* or student*)).tw.<br>4. or/1-3<br>5. suicide/ or suicidal ideation/ or attempted suicide/ or suicidality/ or suicidology/<br>6. suicid*.tw.<br>7. or/5-6<br>8. case report/<br>9. (case report* or case stud*).tw.<br>10. (Book or Authored Book or Edited Book or Dissertation Abstract or Encyclopedia).pt.<br>11. or/8-10<br>12. 4 and 7<br>13. 12 not 11<br><br>Results: **418** citations retrieved
CINAHL (EBSCOHost, 1976-2021)	((MH "Students, Medical") OR (MH "Education, Medical") OR (TI (((medical* or medicine*) N2 (educat* or train* or student*))) OR AB (((medical* or medicine*) N2 (educat* or train* or student*))))) AND (((MH "Suicide") OR (MH "Suicide, Attempted") OR (MH "Suicidal Ideation")) OR (TI suicid* OR AB suicid*))<br><br>Results: **357** citations retrieved
Education Source (EBSCOHost, 1974-2021)	(((DE "Medical education") OR (DE "Medical students" OR DE "Women medical students")) OR (AB ((medical* or medicine*) N2 (educat* or train* or student*)) OR TI ((medical* or medicine*) N2 (educat* or train* or student*)))) AND ((DE "Suicidal behavior in college students") OR (DE "Suicidal behavior in young adults") OR (AB suicid* OR TI suicid*))<br><br>Results: **85** citations retrieved
Scopus	(TITLE-ABS-KEY ((medical* OR medicine*) W/2 (educat* OR train* OR student*)) AND TITLE-ABS-KEY (suicid*)) AND (LIMIT-TO (SRCTYPE , "j")) AND (LIMIT-TO (DOCTYPE , "ar") OR LIMIT-TO (DOCTYPE , "sh"))<br><br>Results: **1,246** citations retrieved

S1 Figure. Forest plots for suicidal ideation risk factors.

a) Academic difficulty

Study or Subgroup	log[Odds Ratio]	SE	Weight	Odds Ratio IV, Random, 95% CI
Torres et al., 2018	0.3772	0.3583	27.3%	1.46 [0.72, 2.94]
Madadin et al., 2020	0.6721	0.2692	37.0%	1.96 [1.16, 3.32]
Pham et al., 2019	0.6981	0.576	14.0%	2.01 [0.65, 6.22]
Desai et al., 2021	1.1939	0.6092	12.8%	3.30 [1.00, 10.89]
Menezes et al., 2012	2.2591	0.7561	8.9%	9.57 [2.18, 42.14]
**Total (95% CI)**			**100.0%**	**2.23 [1.39, 3.58]**

Heterogeneity: Tau² = 0.09; Chi² = 5.69, df = 4 (P = 0.22); I² = 30%
Test for overall effect: Z = 3.32 (P = 0.0009)

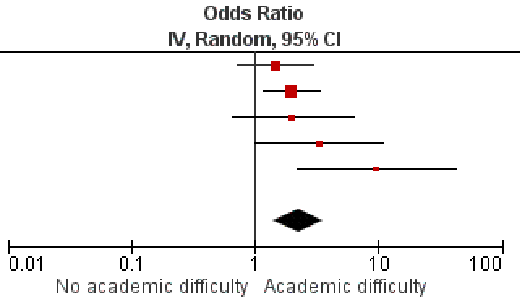

b) Alcohol use

Study or Subgroup	log[Odds Ratio]	SE	Weight	Odds Ratio IV, Random, 95% CI
Torres et al., 2018	-0.4102	0.3939	11.6%	0.66 [0.31, 1.44]
Pham et al., 2019	0.0862	0.3679	12.6%	1.09 [0.53, 2.24]
Jackson et al., 2016	0.1656	0.1084	27.5%	1.18 [0.95, 1.46]
Desalegn et al., 2020	0.4511	0.3702	12.5%	1.57 [0.76, 3.24]
Menezes et al., 2012	0.4818	0.4739	9.0%	1.62 [0.64, 4.10]
Asfaw et al., 2020	0.8154	0.2264	19.9%	2.26 [1.45, 3.52]
Desai et al., 2021	1.1632	0.5637	7.0%	3.20 [1.06, 9.66]
**Total (95% CI)**			**100.0%**	**1.42 [1.02, 1.98]**

Heterogeneity: Tau² = 0.09; Chi² = 12.90, df = 6 (P = 0.04); I² = 54%
Test for overall effect: Z = 2.08 (P = 0.04)

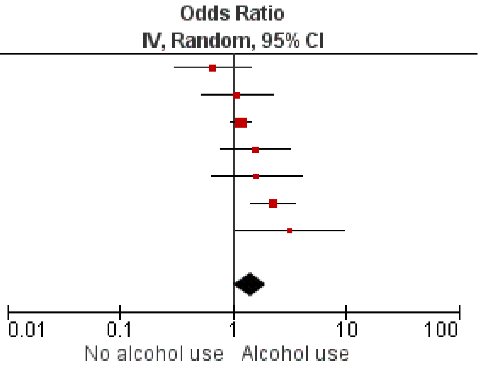

c) Anxiety

Study or Subgroup	log[Odds Ratio]	SE	Weight	Odds Ratio IV, Random, 95% CI
Asfaw et al., 2020	1.0986	0.2384	70.3%	3.00 [1.88, 4.79]
Torres et al., 2018	1.1221	0.3669	29.7%	3.07 [1.50, 6.30]
**Total (95% CI)**			**100.0%**	**3.02 [2.04, 4.47]**

Heterogeneity: Tau² = 0.00; Chi² = 0.00, df = 1 (P = 0.96); I² = 0%
Test for overall effect: Z = 5.53 (P < 0.00001)

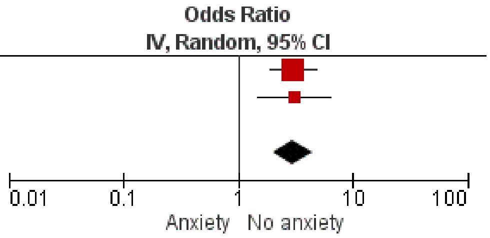

d) Burnout

Study or Subgroup	log[Odds Ratio]	SE	Weight	Odds Ratio IV, Random, 95% CI
Dyrbye et al., 2008	1.4071	0.1571	63.6%	4.08 [3.00, 5.56]
Talih et al., 2018	2.5951	0.6392	36.4%	13.40 [3.83, 46.89]
**Total (95% CI)**			**100.0%**	**6.29 [2.05, 19.30]**

Heterogeneity: Tau² = 0.49; Chi² = 3.26, df = 1 (P = 0.07); I² = 69%
Test for overall effect: Z = 3.22 (P = 0.001)

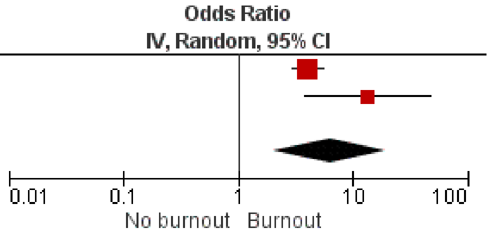

e) Cigarette use

Study or Subgroup	log[Odds Ratio]	SE	Weight	Odds Ratio IV, Random, 95% CI
Desalegn et al., 2020	-0.6349	0.717	16.6%	0.53 [0.13, 2.16]
Desai et al., 2021	-0.1637	0.7563	15.5%	0.85 [0.19, 3.74]
Menezes et al., 2012	0.9927	0.6188	19.9%	2.70 [0.80, 9.08]
Osama et al., 2014	1.1578	0.389	30.4%	3.18 [1.48, 6.82]
Okasha et al., 1981	1.3218	0.6834	17.7%	3.75 [0.98, 14.31]
**Total (95% CI)**			**100.0%**	**1.92 [0.94, 3.92]**

Heterogeneity: Tau² = 0.29; Chi² = 7.14, df = 4 (P = 0.13); I² = 44%
Test for overall effect: Z = 1.79 (P = 0.07)

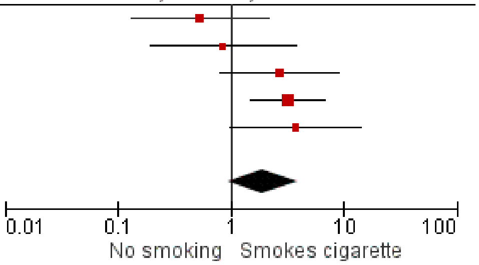

f) Clerkship

Study or Subgroup	log[Odds Ratio]	SE	Weight	Odds Ratio IV, Random, 95% CI
Goebert et al., 2009	0.0834	0.2468	13.7%	1.09 [0.67, 1.76]
Desai et al., 2021	0.1823	0.4377	6.4%	1.20 [0.51, 2.83]
Dyrbye et al., 2008	0.3432	0.136	21.7%	1.41 [1.08, 1.84]
Maser et al., 2019	0.4383	0.1349	21.8%	1.55 [1.19, 2.02]
Tang et al., 2020	0.6626	0.197	17.0%	1.94 [1.32, 2.85]
Ahmed et al., 2016	0.9527	0.3229	9.9%	2.59 [1.38, 4.88]
Menezes et al., 2012	1.1314	0.5015	5.1%	3.10 [1.16, 8.28]
Schwenk et al., 2010	1.7712	0.5604	4.3%	5.88 [1.96, 17.63]
**Total (95% CI)**			**100.0%**	**1.71 [1.33, 2.18]**

Heterogeneity: Tau² = 0.05; Chi² = 14.13, df = 7 (P = 0.05); I² = 50%
Test for overall effect: Z = 4.26 (P < 0.0001)

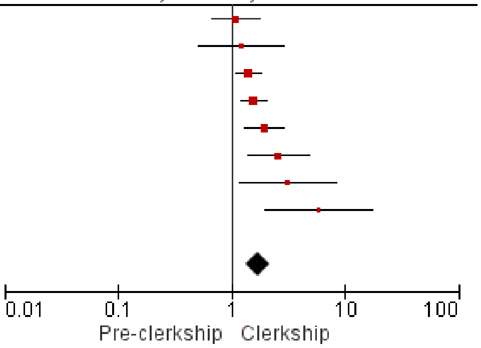

g) Comorbid mental illness

Study or Subgroup	log[Odds Ratio]	SE	Weight	Odds Ratio IV, Random, 95% CI
Desai et al., 2021	0.5306	0.6347	12.9%	1.70 [0.49, 5.90]
Desalegn et al., 2020	0.8755	0.7335	10.9%	2.40 [0.57, 10.11]
Nesan et al., 2020	1.3078	0.6314	13.0%	3.70 [1.07, 12.75]
Osama et al., 2014	1.4221	0.4068	19.4%	4.15 [1.87, 9.20]
Dyrbye et al., 2012	2.2394	0.2093	26.2%	9.39 [6.23, 14.15]
Torres et al., 2018	2.4343	0.4654	17.5%	11.41 [4.58, 28.40]
**Total (95% CI)**			**100.0%**	**5.08 [2.81, 9.18]**

Heterogeneity: Tau² = 0.31; Chi² = 12.81, df = 5 (P = 0.03); I² = 61%
Test for overall effect: Z = 5.37 (P < 0.00001)

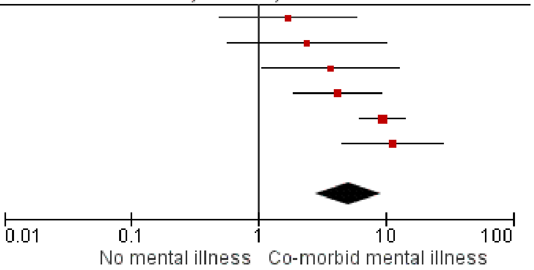

h) Demanding parents

Study or Subgroup	log[Odds Ratio]	SE	Weight	Odds Ratio IV, Random, 95% CI
Ghazanfar et al., 2015	0.561	0.2727	36.4%	1.75 [1.03, 2.99]
Nesan et al., 2020	0.5973	0.4262	14.9%	1.82 [0.79, 4.19]
Osama et al., 2014	0.8599	0.2356	48.7%	2.36 [1.49, 3.75]
**Total (95% CI)**			**100.0%**	**2.04 [1.48, 2.81]**

Heterogeneity: Tau² = 0.00; Chi² = 0.77, df = 2 (P = 0.68); I² = 0%
Test for overall effect: Z = 4.33 (P < 0.0001)

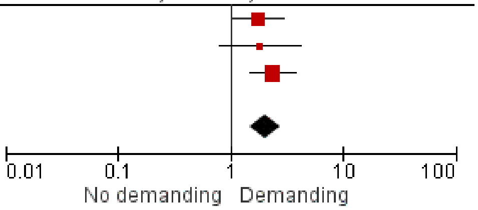

i) Depression

Study or Subgroup	log[Odds Ratio]	SE	Weight	Odds Ratio IV, Random, 95% CI
Asfaw et al., 2020	1.2754	0.2415	14.8%	3.58 [2.23, 5.75]
Schwenk et al., 2010	1.527	0.4542	8.9%	4.60 [1.89, 11.21]
Ghazanfar et al., 2015	1.5614	0.2458	14.6%	4.77 [2.94, 7.71]
Dyrbye et al., 2008	1.8727	0.174	16.9%	6.51 [4.63, 9.15]
Torres et al., 2018	1.8886	0.4493	9.0%	6.61 [2.74, 15.95]
Sobowale et al., 2014	2.1366	0.4321	9.4%	8.47 [3.63, 19.76]
Desalegn et al., 2020	2.3145	0.2029	16.0%	10.12 [6.80, 15.06]
Pham et al., 2019	3.038	0.3889	10.5%	20.86 [9.74, 44.71]
**Total (95% CI)**			**100.0%**	**6.87 [4.80, 9.82]**

Heterogeneity: $Tau^2 = 0.17$; $Chi^2 = 22.32$, $df = 7$ ($P = 0.002$); $I^2 = 69\%$
Test for overall effect: $Z = 10.57$ ($P < 0.00001$)

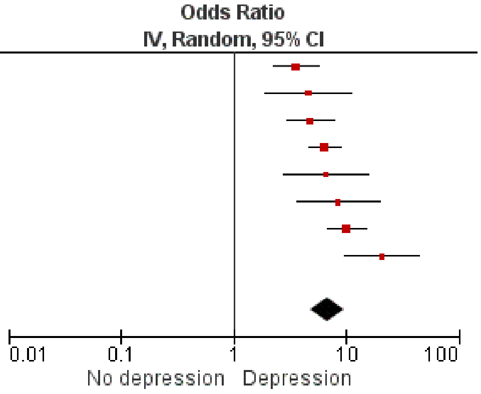

j) Fatigue

Study or Subgroup	log[Odds Ratio]	SE	Weight	Odds Ratio IV, Random, 95% CI
Ghazanfar et al., 2015	0.7638	0.2383	53.5%	2.15 [1.35, 3.42]
Tang et al., 2020	1.8099	0.3646	46.5%	6.11 [2.99, 12.48]
**Total (95% CI)**			**100.0%**	**3.49 [1.26, 9.71]**

Heterogeneity: $Tau^2 = 0.45$; $Chi^2 = 5.77$, $df = 1$ ($P = 0.02$); $I^2 = 83\%$
Test for overall effect: $Z = 2.40$ ($P = 0.02$)

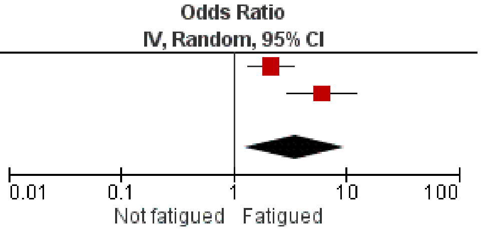

k) Female

Study or Subgroup	log[Odds Ratio]	SE	Weight	Odds Ratio IV, Random, 95% CI
Menezes et al., 2012	-0.429	0.4672	4.3%	0.65 [0.26, 1.63]
Pham et al., 2019	-0.3857	0.4527	4.4%	0.68 [0.28, 1.65]
Compton et al., 2008	-0.2107	0.2777	6.3%	0.81 [0.47, 1.40]
Torres et al., 2018	-0.0149	0.3617	5.3%	0.99 [0.48, 2.00]
Tang et al., 2020	0.0486	0.1964	7.3%	1.05 [0.71, 1.54]
Tareq et al., 2020	0.086	0.2893	6.2%	1.09 [0.62, 1.92]
Dyrbye et al., 2008	0.1205	0.1348	7.9%	1.13 [0.87, 1.47]
Maser et al., 2019	0.1989	0.1276	8.0%	1.22 [0.95, 1.57]
Asfaw et al., 2020	0.27	0.2207	7.0%	1.31 [0.85, 2.02]
Osama et al., 2014	0.3635	0.2372	6.8%	1.44 [0.90, 2.29]
Schwenk et al., 2010	0.4406	0.4669	4.3%	1.55 [0.62, 3.88]
Madadin et al., 2020	0.4952	0.2573	6.6%	1.64 [0.99, 2.72]
Ahmed et al., 2016	0.4955	0.2745	6.4%	1.64 [0.96, 2.81]
Yousaf et al., 2016	0.8214	0.4502	4.4%	2.27 [0.94, 5.49]
Khosravi & Kasaeiyan, 2020	1.4026	0.3163	5.9%	4.07 [2.19, 7.56]
Desalegn et al., 2020	1.6506	0.2148	7.1%	5.21 [3.42, 7.94]
Desai et al., 2021	2.1861	0.8756	1.9%	8.90 [1.60, 49.51]
**Total (95% CI)**			**100.0%**	**1.47 [1.13, 1.92]**

Heterogeneity: $Tau^2 = 0.21$; $Chi^2 = 71.96$, $df = 16$ ($P < 0.00001$); $I^2 = 78\%$
Test for overall effect: $Z = 2.86$ ($P = 0.004$)

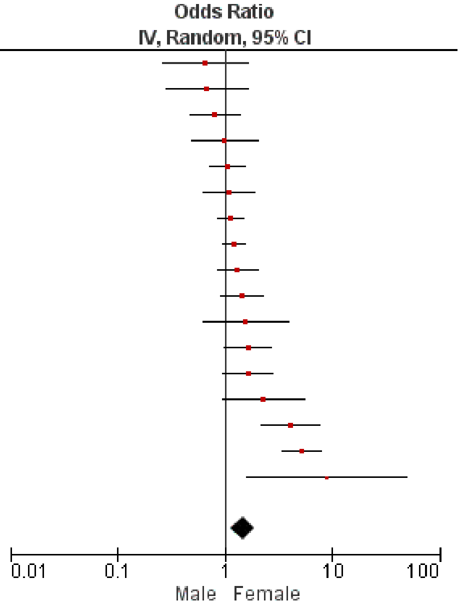

l) Family history of mental illness

Study or Subgroup	log[Odds Ratio]	SE	Weight	Odds Ratio IV, Random, 95% CI
Asfaw et al., 2020	-0.0943	0.3825	31.7%	0.91 [0.43, 1.93]
Desalegn et al., 2020	0.6313	0.4359	28.9%	1.88 [0.80, 4.42]
Ghazanfar et al., 2015	1.0935	0.2441	39.4%	2.98 [1.85, 4.82]
**Total (95% CI)**			**100.0%**	**1.79 [0.86, 3.74]**

Heterogeneity: Tau² = 0.30; Chi² = 6.91, df = 2 (P = 0.03); I² = 71%
Test for overall effect: Z = 1.56 (P = 0.12)

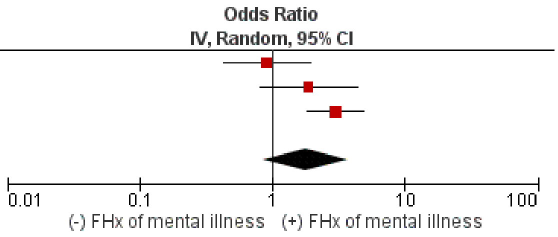

m) Family history of suicide attempt

Study or Subgroup	log[Odds Ratio]	SE	Weight	Odds Ratio IV, Random, 95% CI
Asfaw et al., 2020	0.3436	0.4192	69.9%	1.41 [0.62, 3.21]
Desai et al., 2021	-0.3567	0.6392	30.1%	0.70 [0.20, 2.45]
**Total (95% CI)**			**100.0%**	**1.14 [0.57, 2.27]**

Heterogeneity: Tau² = 0.00; Chi² = 0.84, df = 1 (P = 0.36); I² = 0%
Test for overall effect: Z = 0.38 (P = 0.70)

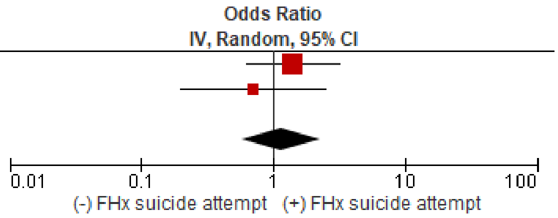

n) Financial burden

Study or Subgroup	log[Odds Ratio]	SE	Weight	Odds Ratio IV, Random, 95% CI
Tang et al., 2020	-0.0225	0.1935	29.1%	0.98 [0.67, 1.43]
Dyrbye et al., 2008	0.3865	0.1671	32.1%	1.47 [1.06, 2.04]
Pham et al., 2019	0.6471	0.2544	22.9%	1.91 [1.16, 3.14]
Ghazanfar et al., 2015	0.7927	0.3473	15.9%	2.21 [1.12, 4.36]
**Total (95% CI)**			**100.0%**	**1.48 [1.06, 2.07]**

Heterogeneity: Tau² = 0.06; Chi² = 6.74, df = 3 (P = 0.08); I² = 55%
Test for overall effect: Z = 2.29 (P = 0.02)

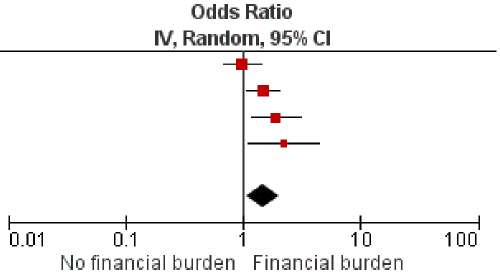

o) History of physical or sexual abuse

Study or Subgroup	log[Odds Ratio]	SE	Weight	Odds Ratio IV, Random, 95% CI
Madadin et al., 2020	0.7602	0.2815	65.9%	2.14 [1.23, 3.71]
Menezes et al., 2012	1.2408	0.5392	18.0%	3.46 [1.20, 9.95]
Desai et al., 2021	1.361	0.5684	16.2%	3.90 [1.28, 11.88]
**Total (95% CI)**			**100.0%**	**2.57 [1.64, 4.02]**

Heterogeneity: Tau² = 0.00; Chi² = 1.27, df = 2 (P = 0.53); I² = 0%
Test for overall effect: Z = 4.13 (P < 0.0001)

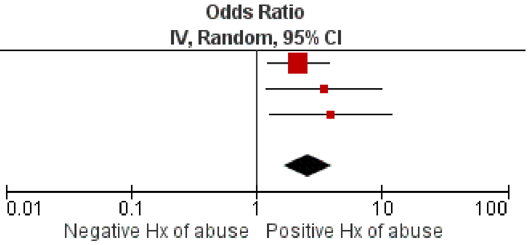

p) History of physical assault

Study or Subgroup	log[Odds Ratio]	SE	Weight	Odds Ratio IV, Random, 95% CI
Nesan et al., 2020	1.0355	0.4697	45.6%	2.82 [1.12, 7.07]
Osama et al., 2014	1.3242	0.4298	54.4%	3.76 [1.62, 8.73]
**Total (95% CI)**			**100.0%**	**3.30 [1.77, 6.14]**

Heterogeneity: Tau² = 0.00; Chi² = 0.21, df = 1 (P = 0.65); I² = 0%
Test for overall effect: Z = 3.76 (P = 0.0002)

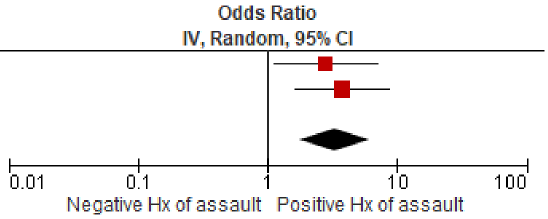

q) Living alone

Study or Subgroup	log[Odds Ratio]	SE	Weight	Odds Ratio IV, Random, 95% CI
Pham et al., 2019	-0.2485	0.8082	8.3%	0.78 [0.16, 3.80]
Nesan et al., 2020	0.849	0.301	59.6%	2.34 [1.30, 4.22]
Torres et al., 2018	0.8713	0.41	32.1%	2.39 [1.07, 5.34]
**Total (95% CI)**			**100.0%**	**2.15 [1.36, 3.39]**

Heterogeneity: Tau² = 0.00; Chi² = 1.72, df = 2 (P = 0.42); I² = 0%
Test for overall effect: Z = 3.29 (P = 0.0010)

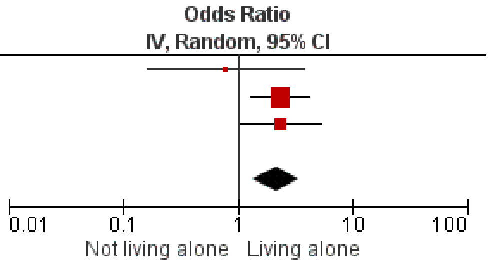

r) Living away from home

Study or Subgroup	log[Odds Ratio]	SE	Weight	Odds Ratio IV, Random, 95% CI
Pham et al., 2019	-0.1374	0.4155	20.0%	0.87 [0.39, 1.97]
Nesan et al., 2020	0.849	0.301	20.8%	2.34 [1.30, 4.22]
Desai et al., 2021	0.8755	0.6287	18.2%	2.40 [0.70, 8.23]
Torres et al., 2018	1.2242	0.3673	20.4%	3.40 [1.66, 6.99]
Ahmed et al., 2016	3.6207	0.3355	20.6%	37.36 [19.36, 72.12]
**Total (95% CI)**			**100.0%**	**3.68 [0.98, 13.84]**

Heterogeneity: Tau² = 2.11; Chi² = 61.65, df = 4 (P < 0.00001); I² = 94%
Test for overall effect: Z = 1.93 (P = 0.05)

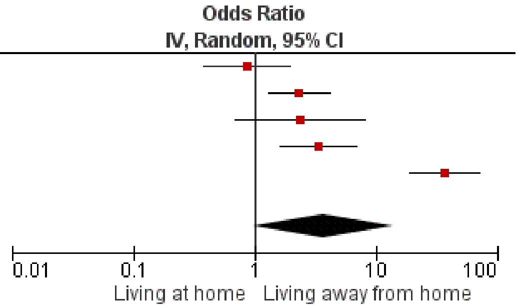

s) Parental neglect

Study or Subgroup	log[Odds Ratio]	SE	Weight	Odds Ratio IV, Random, 95% CI
Madadin et al., 2020	-0.0564	0.3383	30.1%	0.95 [0.49, 1.83]
Menezes et al., 2012	1.2513	0.6342	20.6%	3.49 [1.01, 12.11]
Osama et al., 2014	1.3638	0.3667	29.1%	3.91 [1.91, 8.02]
Nesan et al., 2020	1.4441	0.6454	20.3%	4.24 [1.20, 15.02]
**Total (95% CI)**			**100.0%**	**2.53 [1.08, 5.92]**

Heterogeneity: Tau² = 0.51; Chi² = 10.26, df = 3 (P = 0.02); I² = 71%
Test for overall effect: Z = 2.15 (P = 0.03)

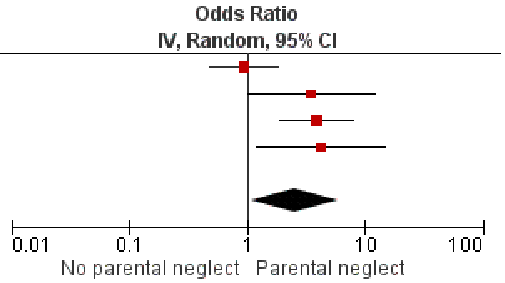

t) Poor social support

Study or Subgroup	log[Odds Ratio]	SE	Weight	Odds Ratio IV, Random, 95% CI
Desalegn et al., 2020	0.6678	0.5451	17.3%	1.95 [0.67, 5.68]
Asfaw et al., 2020	0.9439	0.3063	32.4%	2.57 [1.41, 4.68]
Torres et al., 2018	0.9567	0.3606	28.0%	2.60 [1.28, 5.28]
Ghazanfar et al., 2015	2.0606	0.4467	22.3%	7.85 [3.27, 18.84]
**Total (95% CI)**			**100.0%**	**3.15 [1.84, 5.41]**

Heterogeneity: Tau² = 0.14; Chi² = 5.66, df = 3 (P = 0.13); I² = 47%
Test for overall effect: Z = 4.17 (P < 0.0001)

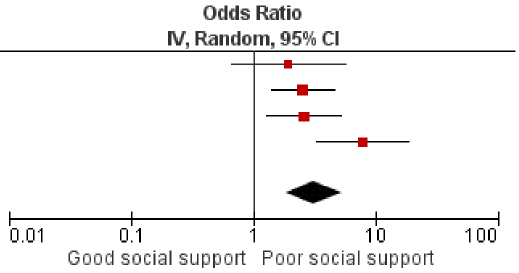

u) Ragged by others

Study or Subgroup	log[Odds Ratio]	SE	Weight	Odds Ratio IV, Random, 95% CI
Osama et al., 2014	0.9163	0.4378	67.3%	2.50 [1.06, 5.90]
Menezes et al., 2012	1.1585	0.6283	32.7%	3.19 [0.93, 10.91]
**Total (95% CI)**			**100.0%**	**2.71 [1.34, 5.47]**

Heterogeneity: Tau² = 0.00; Chi² = 0.10, df = 1 (P = 0.75); I² = 0%
Test for overall effect: Z = 2.77 (P = 0.006)

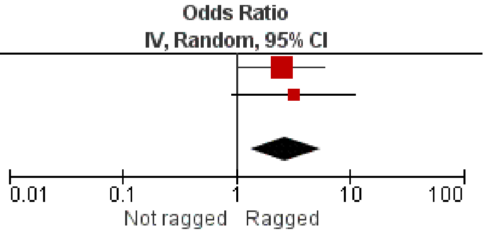

v) Sleep difficulty

Study or Subgroup	Difficulty sleeping Events	Difficulty sleeping Total	No sleep difficulty Events	No sleep difficulty Total	Weight	Odds Ratio IV, Random, 95% CI
Ghazanfar et al., 2015	15	121	62	1011	56.2%	2.17 [1.19, 3.94]
Okasha et al., 1981	25	30	41	102	43.8%	7.44 [2.63, 21.02]
**Total (95% CI)**		**151**		**1113**	**100.0%**	**3.72 [1.12, 12.35]**
Total events	40		103			
Heterogeneity: Tau² = 0.57; Chi² = 4.07, df = 1 (P = 0.04); I² = 75%						
Test for overall effect: Z = 2.15 (P = 0.03)						

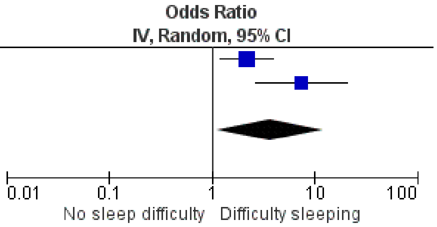

w) Stress

Study or Subgroup	log[Odds Ratio]	SE	Weight	Odds Ratio IV, Random, 95% CI
Asfaw et al., 2020	0.2546	0.2128	27.2%	1.29 [0.85, 1.96]
Menezes et al., 2012	1.1585	0.6283	19.8%	3.19 [0.93, 10.91]
Compton et al., 2008	1.2326	0.1865	27.5%	3.43 [2.38, 4.94]
Tang et al., 2020	2.6519	0.3315	25.5%	14.18 [7.41, 27.16]
**Total (95% CI)**			**100.0%**	**3.72 [1.39, 9.94]**
Heterogeneity: Tau² = 0.88; Chi² = 38.21, df = 3 (P < 0.00001); I² = 92%				
Test for overall effect: Z = 2.62 (P = 0.009)				

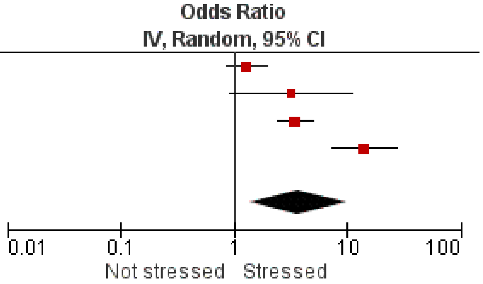

x) Substance abuse

Study or Subgroup	log[Odds Ratio]	SE	Weight	Odds Ratio IV, Random, 95% CI
Jackson et al., 2016	0.1656	0.1084	34.8%	1.18 [0.95, 1.46]
Desai et al., 2021	0.47	0.7335	25.4%	1.60 [0.38, 6.74]
Menezes et al., 2012	2.2083	1.0274	20.0%	9.10 [1.21, 68.17]
Osama et al., 2014	3.3516	1.0421	19.8%	28.55 [3.70, 220.10]
**Total (95% CI)**			**100.0%**	**3.60 [0.91, 14.25]**
Heterogeneity: Tau² = 1.40; Chi² = 13.14, df = 3 (P = 0.004); I² = 77%				
Test for overall effect: Z = 1.83 (P = 0.07)				

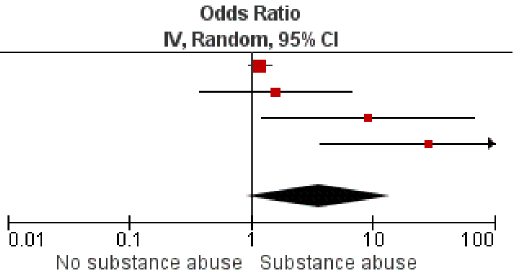

y) Thoughts of dropping out

Study or Subgroup	log[Odds Ratio]	SE	Weight	Odds Ratio IV, Random, 95% CI
Torres et al., 2018	1.0296	0.4115	23.9%	2.80 [1.25, 6.27]
Ghazanfar et al., 2015	1.0913	0.2658	57.2%	2.98 [1.77, 5.01]
Schwenk et al., 2010	1.2321	0.4619	18.9%	3.43 [1.39, 8.48]
**Total (95% CI)**			**100.0%**	**3.01 [2.03, 4.47]**
Heterogeneity: Tau² = 0.00; Chi² = 0.11, df = 2 (P = 0.95); I² = 0%				
Test for overall effect: Z = 5.49 (P < 0.00001)				

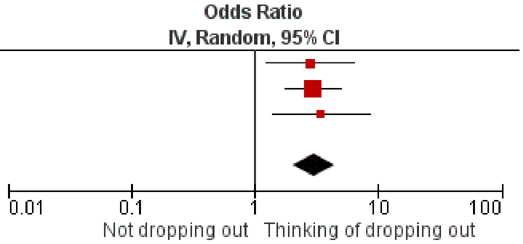

S2 Figure. Forest plots for suicide attempt risk factors.

a) Alcohol use

Study or Subgroup	log[Odds Ratio]	SE	Weight	Odds Ratio IV, Random, 95% CI
Asfaw et al., 2020	0.207	0.4492	79.6%	1.23 [0.51, 2.97]
Desalegn et al., 2020	0.8713	0.8872	20.4%	2.39 [0.42, 13.60]
**Total (95% CI)**			**100.0%**	**1.41 [0.64, 3.09]**

Heterogeneity: $Tau^2 = 0.00$; $Chi^2 = 0.45$, df = 1 (P = 0.50); $I^2 = 0\%$
Test for overall effect: Z = 0.85 (P = 0.39)

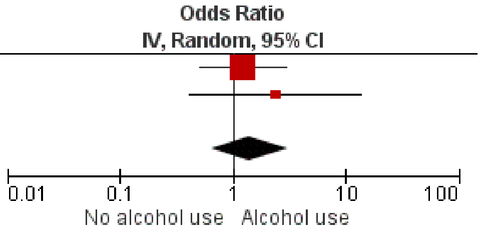

b) Depression

Study or Subgroup	log[Odds Ratio]	SE	Weight	Odds Ratio IV, Random, 95% CI
Asfaw et al., 2020	1.6864	0.6708	4.5%	5.40 [1.45, 20.11]
Desalegn et al., 2020	2.3665	0.1458	95.5%	10.66 [8.01, 14.19]
**Total (95% CI)**			**100.0%**	**10.34 [7.82, 13.67]**

Heterogeneity: $Tau^2 = 0.00$; $Chi^2 = 0.98$, df = 1 (P = 0.32); $I^2 = 0\%$
Test for overall effect: Z = 16.39 (P < 0.00001)

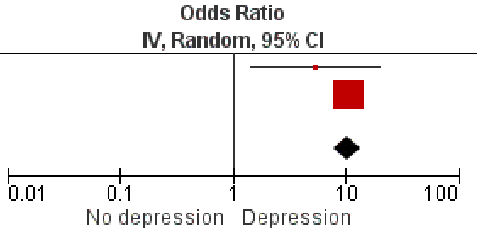

c) Female

Study or Subgroup	log[Odds Ratio]	SE	Weight	Odds Ratio IV, Random, 95% CI
Tang et al., 2020	0.475	0.6947	26.6%	1.61 [0.41, 6.28]
Asfaw et al., 2020	0.6313	0.411	34.1%	1.88 [0.84, 4.21]
Desalegn et al., 2020	2.0894	0.1485	39.3%	8.08 [6.04, 10.81]
**Total (95% CI)**			**100.0%**	**3.20 [0.95, 10.71]**

Heterogeneity: $Tau^2 = 0.95$; $Chi^2 = 15.30$, df = 2 (P = 0.0005); $I^2 = 87\%$
Test for overall effect: Z = 1.88 (P = 0.06)

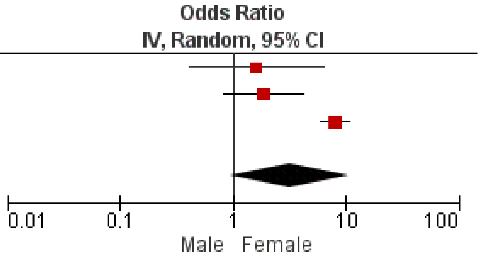

d) Family history of suicidal behaviour (*FHx of committed suicide)

Study or Subgroup	log[Odds Ratio]	SE	Weight	Odds Ratio IV, Random, 95% CI
Asfaw et al., 2020	0.5247	0.5916	60.4%	1.69 [0.53, 5.39]
Asfaw et al., 2020*	0.7178	0.7302	39.6%	2.05 [0.49, 8.58]
**Total (95% CI)**			**100.0%**	**1.82 [0.74, 4.49]**

Heterogeneity: $Tau^2 = 0.00$; $Chi^2 = 0.04$, df = 1 (P = 0.84); $I^2 = 0\%$
Test for overall effect: Z = 1.31 (P = 0.19)

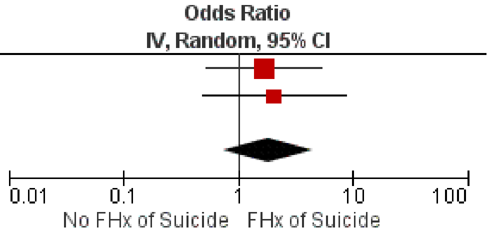

e) Stress

Study or Subgroup	log[Odds Ratio]	SE	Weight	Odds Ratio IV, Random, 95% CI
Asfaw et al., 2020	0.3784	0.4208	54.3%	1.46 [0.64, 3.33]
Tang et al., 2020	2.1269	0.6627	45.7%	8.39 [2.29, 30.75]
**Total (95% CI)**			**100.0%**	**3.25 [0.59, 17.90]**

Heterogeneity: $Tau^2 = 1.22$; $Chi^2 = 4.96$, df = 1 (P = 0.03); $I^2 = 80\%$
Test for overall effect: Z = 1.35 (P = 0.18)

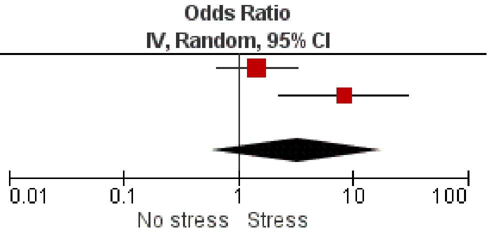

S3 Figure. Funnel plot of studies reporting female gender as a SI risk factor.

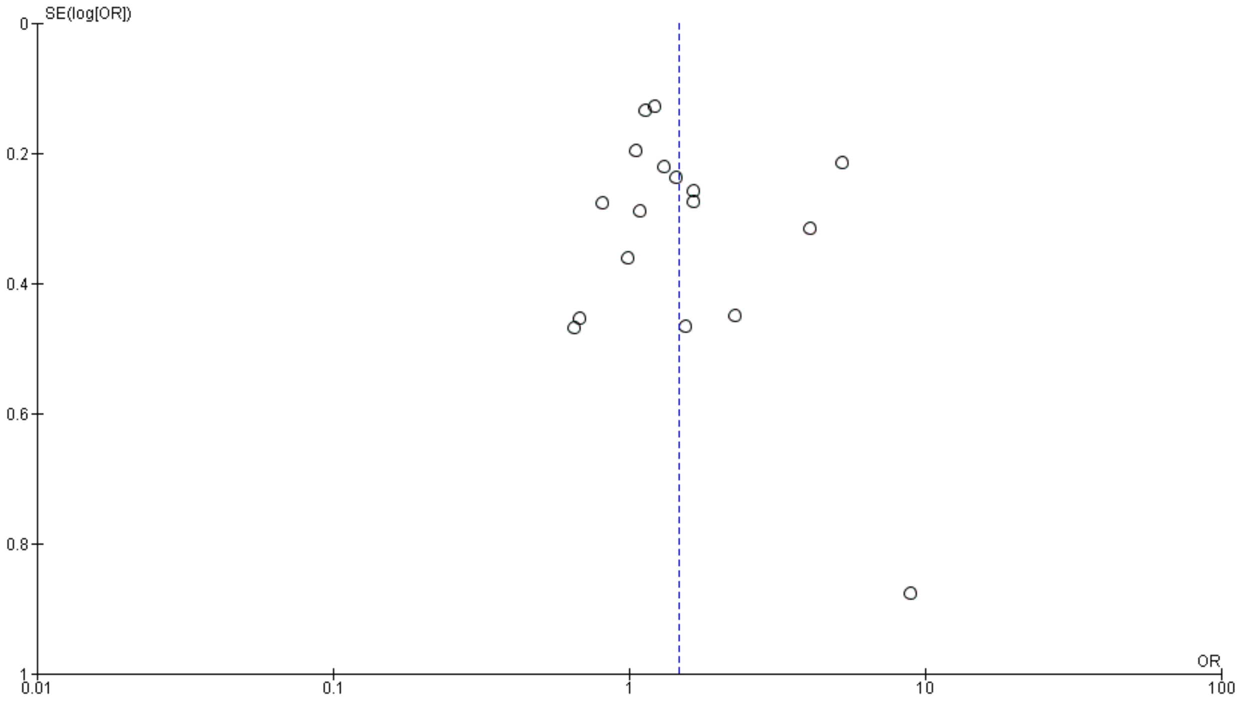

Supplement: S1 File — (PDF) [file pone.0261785.s006.pdf]
